# Evolutionary Timeline and Genomic Plasticity Underlying the Lifestyle Diversity in *Rhizobiales*

**DOI:** 10.1128/mSystems.00438-20

**Published:** 2020-07-14

**Authors:** Sishuo Wang, Andrew Meade, Hon-Ming Lam, Haiwei Luo

**Affiliations:** a School of Life Sciences and State Key Laboratory of Agrobiotechnology, The Chinese University of Hong Kong, Shatin, Hong Kong SAR; b School of Biological Sciences, University of Reading, Whiteknights, Reading, United Kingdom; c Shenzhen Research Institute, The Chinese University of Hong Kong, Shenzhen, China; University of California, San Diego

**Keywords:** *Rhizobiales*, rhizobia, lifestyle evolution, molecular clock, bacterial evolution, microbial evolution, molecular dating, symbiosis

## Abstract

Members of the order *Rhizobiales* include those capable of nitrogen fixation in nodules as well as pathogens of animals and plants. This lifestyle diversity has important implications for agricultural and medical research. Leveraging large-scale genomic data, we infer that *Rhizobiales* originated as a free-living ancestor ∼1,500 million years ago (Mya) and that the later emergence of host-associated lifestyles broadly coincided with the rise of their eukaryotic hosts. In particular, the first nodulating lineage arose from either *Azorhizobium* or *Bradyrhizobium* 150 to 80 Mya, a time range in general concurrent with the emergence of legumes. The rates of lifestyle transitions are highly variable; nodule association is more likely to be lost than gained, whereas animal association likely represents an evolutionary dead end. We searched for statistical correlations between gene presence and lifestyle and identified genes likely contributing to the transition and adaptation to the same lifestyle in divergent lineages. Among the genes potentially promoting successful transitions to major nodulation lineages, the *nod* and *nif* clusters for nodulation and nitrogen fixation, respectively, were repeatedly acquired during each transition; the *fix*, *dct*, and *phb* clusters involved in energy conservation under micro-oxic conditions were present in the nonnodulating ancestors; and the secretion systems were acquired in lineage-specific patterns. Our study data suggest that increased eukaryote diversity drives lifestyle diversification of bacteria and highlight both acquired and preexisting traits facilitating the origin of host association.

**IMPORTANCE** Bacteria form diverse interactions with eukaryotic hosts. This is well represented by the *Rhizobiales*, a clade of *Alphaproteobacteria* strategically important for their large diversity of lifestyles with implications for agricultural and medical research. To investigate their lifestyle evolution, we compiled a comprehensive data set of genomes and lifestyle information for over 1,000 *Rhizobiales* genomes. We show that the origins of major host-associated lineages in *Rhizobiales* broadly coincided with the emergences of their host plants/animals, suggesting bacterium-host interactions as a driving force in the evolution of *Rhizobiales*. We further found that, in addition to gene gains, preexisting traits and recurrent losses of specific genomic traits may have played underrecognized roles in the origin of host-associated lineages, providing clues to genetic engineering of microbial agricultural inoculants and prevention of the emergence of potential plant/animal pathogens.

## Abstract (precis)

Bacteria form diverse interactions with eukaryotic hosts. This is well represented by the *Rhizobiales*, a clade of *Alphaproteobacteria* strategically important for their large diversity of lifestyles with implications for agricultural and medical research. To investigate their lifestyle evolution, we compiled a comprehensive data set of genomes and lifestyle information for over 1,000 *Rhizobiales* genomes. We show that the origins of major host-associated lineages in *Rhizobiales* broadly coincided with the emergences of their host plants/animals, suggesting bacterium-host interactions as a driving force in the evolution of *Rhizobiales*. We further found that, in addition to gene gains, preexisting traits and recurrent losses of specific genomic traits may have played underrecognized roles in the origin of host-associated lineages, providing clues to genetic engineering of microbial agricultural inoculants and prevention of the emergence of potential plant/animal pathogens.

## INTRODUCTION

Bacteria form diverse interactions with their hosts, which, from commensalism to mutualism and from parasitism to pathogenesis, have played an important role in the emergence of complex life forms and many other evolutionary and ecological processes (1–4). The diversity of lifestyles in bacteria is well epitomized by the alphaproteobacterial order *Rhizobiales* (5–8). A large proportion of *Rhizobiales* species adapt to host-associated lifestyles, many of which have important agricultural, ecological, and medical implications, making *Rhizobiales* an ideal system to investigate the evolution of bacterial lifestyles (5, 6). This lifestyle diversity includes rhizobia, which form nodules and establish a symbiotic relationship with legumes; plant pathogens such as the causative agents of root tumor *Agrobacterium*; and *Bartonella* and *Brucella*, two important alphaproteobacterial animal pathogens colonizing the erythrocytes of mammals.

In ancient times, bacteria primarily adapted to a free-living lifestyle occupying aquatic and terrestrial habitats (9). Alongside the geosphere-biosphere interactions that have occurred since the Cambrian explosion, the past 500 million years (My) have witnessed a massive diversification of animals and plants (10). This might create many opportunities for bacteria to interact with diverse eukaryotes and might facilitate the radiation of bacteria (5, 11). However, to our knowledge, the time frames of the coevolution between *Rhizobiales* and their hosts have never been determined. This leaves open two important issues: (i) establishing from which lineages the first nodulating organisms and animal pathogens originated and (ii) determining how the establishment of successful bacterial symbiosis is driven by their coevolution with eukaryotes.

Adaptations to diverse lifestyles suggest high genomic plasticity of *Rhizobiales* (12, 13). Intriguingly, species of the same lifestyle may not form monophyletic groups in *Rhizobiales* (6, 14). Recurrent gains and losses of genes important to specific lifestyles may make independent lifestyle transitions occur (6, 15, 16). Despite extensive genetic and physiological studies, our knowledge of these genes, however, is still limited. A genome-wide computational identification of the genes associated with each lifestyle which circumvents the low-throughput limitations of wet-lab experiments is urgently needed. By tracing the evolution of lifestyle-correlated genes during lifestyle transitions, we can better understand how rhizobia and other host-associated bacteria evolve step by step at the genomic level.

Despite the many important issues raised regarding lifestyle diversity in *Rhizobiales*, genomics studies have mainly focused on organisms with a given lifestyle (13, 17, 18), with the transitions between lifestyles awaiting further exploration. In the present study, we compiled comprehensive *Rhizobiales* genomics data sets, reconstructed a timeline of the evolutionary origins of distinct host-associated lifestyles, linked them to the evolutionary time of their hosts, and explored the functional changes underlying the transitions between lifestyles. We highlighted the roles of increased diversity of eukaryotes and genomic plasticity in the evolution of host-associated bacteria in *Rhizobiales*.

## RESULTS AND DISCUSSION

### Lifestyle diversification in *Rhizobiales*.

**FIG 1:**
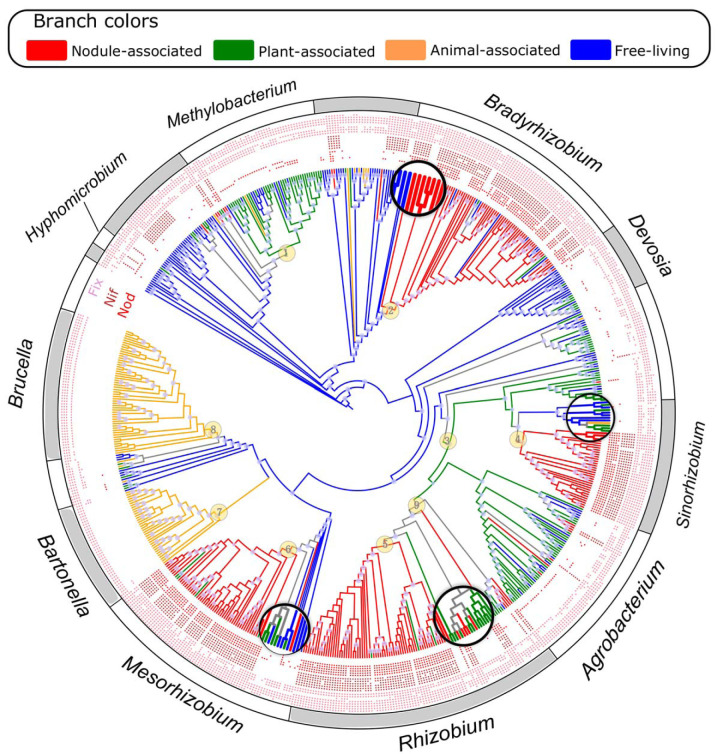
Ancestral lifestyle reconstruction of the *Rhizobiales*. Ancestral lifestyles were inferred using the parsimony method in Mesquite. Branches in red, green, orange, and blue indicate nodule-associated, plant-associated, animal-associated, and free-living lifestyles, respectively. Numbered nodes represent the origins of the host-associated lifestyles (the nodule-associated, plant-associated, and animal-associated lifestyles). Zoomed-in lineages represent species that split early within the *Bradyrhizobium*, *Mesorhizobium*, *Rhizobium*, and *Sinorhizobium*. The three layers are each indicated with a distinct color surrounding the phylogeny to denote the presence/absence of key nodule-related genes (the *nod*, *nif*, and *fix* genes). The genes selected to represent each pathway/complex are shown in Text S1. Purple circles on the phylogeny represent nodes supported by IQ-Tree ultrafast bootstrap values of ≥95%. The outgroups are not shown.

The *Rhizobiales* taxa fall into four lifestyles, the nodule-associated, plant-associated, animal-associated, and free-living lifestyles. We inferred ancestral lifestyles of *Rhizobiales* using the maximum parsimony method implemented in Mesquite based upon the lifestyles of extant taxa (see Data Set S1 in the supplemental material for the complete list) and on their concatenated ribosomal protein phylogenies (see Materials and Methods). We did not use the maximum likelihood method in the main analysis because the short branches across the phylogeny can lead to overestimations of transition rates and thus to inaccurate ancestral lifestyle inferences (19), although we included a maximum likelihood analysis to assess the consistency of the results (see below). According to this procedure, the last common ancestor (LCA) of *Rhizobiales* was likely a free-living bacterium (Fig. 1), consistent with the fact that a vast majority of the branches of *Rhizobiales* that split early are represented by free-living members (Fig. 1). The nodule-associated lifestyle evolved multiple times, during which four major origins led to the formation of four well-known rhizobia genera, namely, *Bradyrhizobium*, *Mesorhizobium*, *Rhizobium*, and *Sinorhizobium*/*Ensifer* (nodes 2, 6, 5, and 4, respectively, in Fig. 1). Interestingly, none of the basal groups of these four rhizobia genera include nodule-associated bacteria: the basal members of *Bradyrhizobium* are free-living bacteria, those of *Rhizobium* are plant associates, and those of *Sinorhizobium* and *Mesorhizobium* take plant-associated or free-living lifestyles (Fig. 1). This suggests that the nodule-associated lifestyle in all of the four genera evolved relatively recently within each genus.

**FIG 2:**
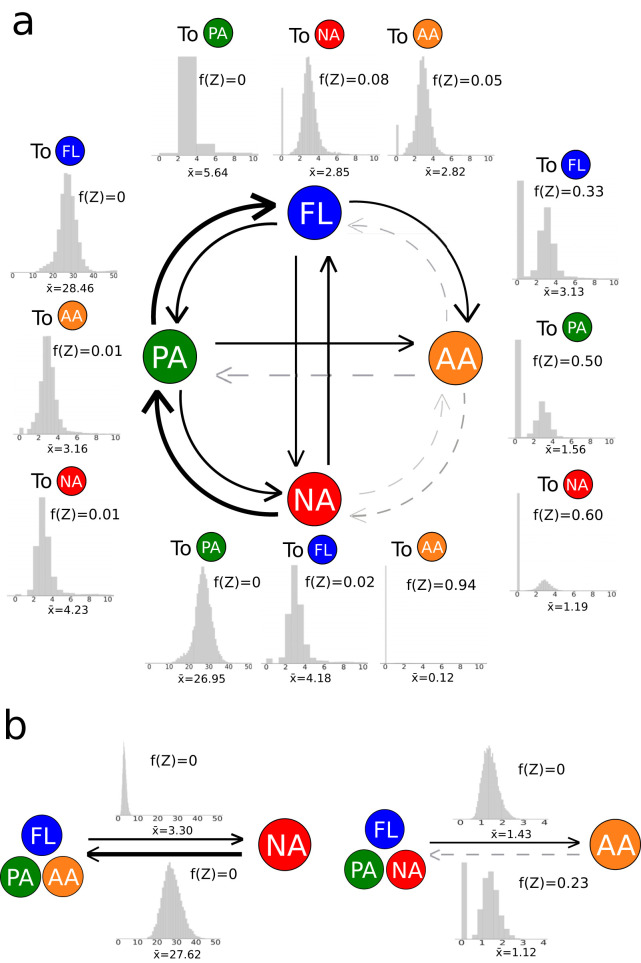
Rates of transitions between lifestyles. The width of each arrow is proportional to the transition rate (log transformed). Gray dashed lines denote transition rates that are not significantly different from zero (logBF < 2). The posterior distributions of transition rates derived by the reversible-jump MCMC method are shown as histograms. The means of transition rates and Z-scores are displayed adjacent to the histogram. Expressed as percentages, Z-values represent the proportions of sampled runs in the Markov chain where the transition rate is assigned to be zero. Z-values allow examination of the contingency of evolutionary transitions, with higher values signifying lifestyle transitions that are less likely to occur in evolution. Nodule-associated, plant-associated, animal-associated, and free-living lifestyles are abbreviated as NA, PA, AA, and FL, respectively. The color that represents each lifestyle is the same as that defined for Fig. 1. (a) Pairwise comparisons of transition rates between lifestyles. (b) Comparisons of transition rates between nodule-associated lifestyles (NA) and non-nodule-associated lifestyles (AA, PA, and FL) and between animal-associated lifestyles (AA) and non-animal-associated lifestyles (NA, PA, and FL).

**FIG 3:**
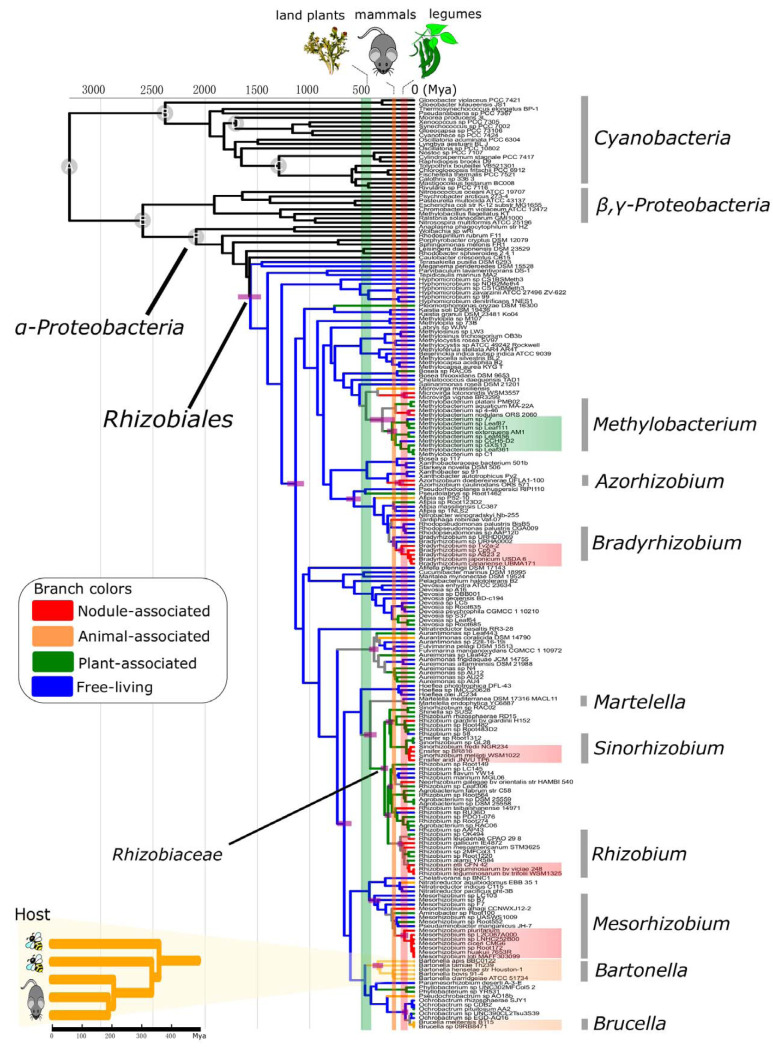
Time tree of the *Rhizobiales* with selected taxa from *Proteobacteria* as the outgroup. Divergence time was estimated using MCMCTree on the species phylogeny shown in Fig. 1 (performed with only the 176 representative species). Nodes marked with a gray circle represent the calibration points in *Proteobacteria* and cyanobacteria (see Text S1 for details). Branch colors are based on the ancestral lifestyle reconstruction shown in Fig. 1. Vertical bars in green, orange, and red indicate the origin times of the primary hosts of extant *Rhizobiales*, namely, land plants, mammals, and legumes, respectively. The panel in the lower left corner of the figure shows a zoomed-in view of the evolutionary timeline of *Bartonella*, where the hosts are also shown next to the tips of the phylogeny. Node bars denote the 95% HPD interval of posterior dates (for key nodes related to the origins of host association only).

**FIG 4:**
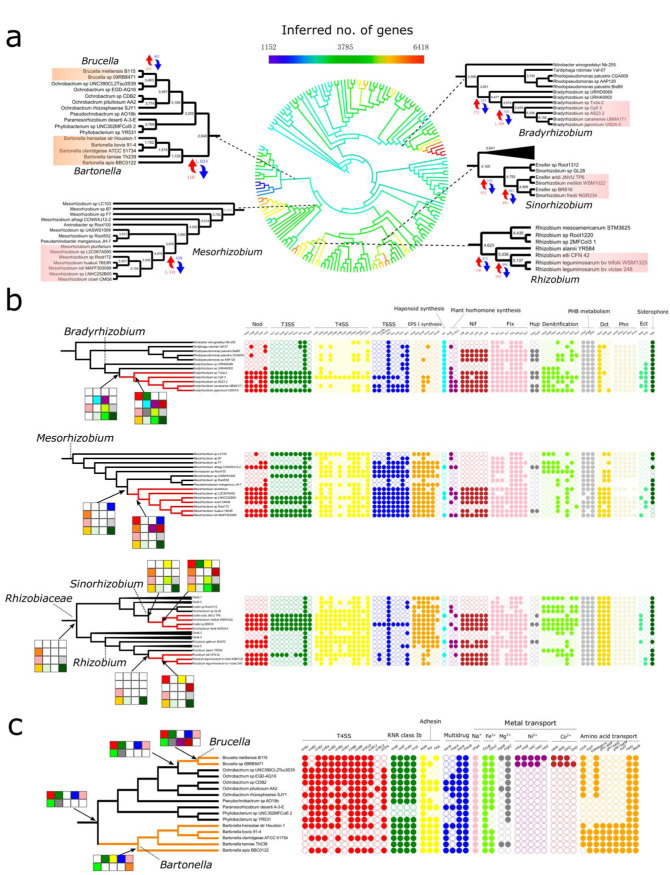
Ancestral genome reconstruction and gene gains/losses during the evolution of *Rhizobiales*. (a) Genome size evolution in the *Rhizobiales*. The colors of the branches in the phylogeny represent the changes of gene numbers in the genomes. Ancestral genomes were inferred with BadiRate based on gene family clustering using OrthoFinder. (b and c) The phyletic pattern and inferred gain/loss of lifestyle-correlated genes in the evolution of nodule-associated lineages (b) and animal-associated lineages (c). Branches in red and orange indicate nodulating and animal-associated lineages, respectively. Selected pathways/complexes important to the host-associated lifestyle are shown next to the trees. Genes were selected based on the criteria detailed in Text S1. Solid and open circles denote the presence and absence of the corresponding genes, respectively. Presence/absence at ancestral nodes inferred by BadiRate is indicated adjacent to the node. Pathways or complexes with ≥50% or <50% of genes present are indicated by a solid or open square adjacent to the ancestral nodes, respectively, based on the reconstruction of ancestral genome content.

10.1128/mSystems.00438-20.10 DATA SET S1 (a) Lifestyle and genome information for all 1,264 *Rhizobiales* genomes downloaded from the NCBI RefSeq database (July 2017). Among them, a total of 655 genomes were analyzed in the study (column F), referred to as “the full set of genomes.” The genomes in this set were used in phylogenomic tree construction (Fig. 1), ancestral lifestyle inference (Fig. 1), lifestyle transition rate analysis (Fig. 2), and identification of lifestyle-associated genes. A set of 176 representative genomes of their OTUs (column G) were used in molecular dating (Fig. 3) and gene gain/loss analysis (Fig. 4). The reference genomes are those commonly used in academics and industry (column H) and were preferentially selected as the OTU representatives (see Materials and Methods). The source(s) of the isolation site of each genome, if available, is derived from the NCBI BioSample database, other public databases, or the literature (references are shown at the end). (b) The full list of lifestyle-correlated gene families that belong to the functional categories/pathways/complexes described in the main text. (c to f) Gene families showing significant correlation with the corresponding lifestyle with the full set of 655 genomes using BayesTraits. The details of the methods used for identification of the lifestyle-correlated gene families are provided in Materials and Methods. Only those with a *q* value (adjusted *P* value, Bonferroni correction) below 0.05 and rate differences above zero are displayed. (c) Nodule-associated (NA) lifestyle. (d) Animal-associated (AA) lifestyle. (e) Plant-associated (PA) lifestyle. (f) Free-living (FL) lifestyle. Download Data Set S1, XLSX file, 0.7 MB. Copyright © 2020 Wang et al. 2020 Wang et al. This content is distributed under the terms of the Creative Commons Attribution 4.0 International license.

10.1128/mSystems.00438-20.1 TEXT S1 (a) Supplemental discussion. (b) Selection of genes included in Fig. 1 and 4. (c) Calibration information. Download Text S1, DOCX file, 0.1 MB. Copyright © 2020 Wang et al. 2020 Wang et al. This content is distributed under the terms of the Creative Commons Attribution 4.0 International license.

Animal associates showed two major independent origins leading to *Bartonella* (node 7 in Fig. 1) and *Brucella* (node 8 in Fig. 1). Since some of their close relatives, *Ochrobactrum*, for example, are increasingly recognized as opportunistic pathogens, there is a chance that their common ancestor had already adapted to an animal-associated lifestyle. We also identified two major origins of plant-associated members: one within the *Methylobacterium* (node 1 in Fig. 1) and the other corresponding to the LCA of the *Rhizobiaceae* (node 3 in Fig. 1). Species from the *Rhizobiaceae* further diversified into nodulating members of *Rhizobium* and *Sinorhizobium*, while others, in particular, those from *Agrobacterium*, plausibly retained the ancestral plant-associated lifestyle (and evolved plant pathogens later [17]) (Fig. 1). It is possible that a few host-associated lineages have a deeper origin, including *Rhizobiaceae*, where nodulating members exhibited mosaic distributions in phylogeny (Fig. 1), implying a more complex evolutionary history. We therefore updated the analysis by inclusion of the recently available genomes of a large number (>300) of *Rhizobiaceae* isolates from the roots of several nonlegume plants (8). Our updated phylogenomic tree showed that the phylogenetic positions of the major group of the nodulating *Rhizobium* (see Fig. S1a in the supplemental material) and of the nodulating *Sinorhizobium* (Fig. S1b) remained congruent with those shown in Fig. 1, suggesting the robustness of ancestral lifestyle reconstruction in these lineages.

10.1128/mSystems.00438-20.2 FIG S1 The phylogeny of *Rhizobium* (a) and *Sinorhizobium* (b), including isolates sequenced from a recent study (8). The phylogenetic tree was constructed based on the concatenated alignment of ribosomal proteins. Purple circles on the phylogeny represent the nodes that are supported by IQ-Tree ultrafast bootstrap values of at least 95%. The layer adjacent to the taxon name denotes different lifestyles, with red, green, orange, and blue representing nodule-associated, plant-associated, animal-associated, and free-living lifestyle, respectively (as shown in Fig. 1). The black circles in the next layer denote those sequenced in a more recent study (8). The red star indicates the origin of nodule association inferred as described for Fig. 1. Download FIG S1, PDF file, 0.1 MB. Copyright © 2020 Wang et al. 2020 Wang et al. This content is distributed under the terms of the Creative Commons Attribution 4.0 International license.

To validate the origins of the host-associated lifestyles with the parsimony-based approach described above, we applied the maximum likelihood method implemented in BayesTraits (see Materials and Methods). Some of the host associations were estimated to have a deeper origin (nodes 1, 3, and 4 in Fig. 1), but the general pattern did not change (Fig. S2a). We further assessed the impacts of taxon sampling (Fig. S2b) and the set of genes used to build phylogeny (Fig. S2c) on lifestyle reconstruction. In both cases, the reconstructed lifestyles are in good agreement with the data in Fig. 1.

10.1128/mSystems.00438-20.3 FIG S2 Ancestral lifestyle reconstruction using alternative strategies. (a) Lifestyles predicted using the maximum likelihood method with BayesTraits. (b) Lifestyles predicted using the maximum parsimony method with a broader taxon sampling, i.e., without removing genomes with completeness below 99%. (c) Lifestyles predicted using the maximum parsimony method based on the phylogeny constructed with 101 single-copy genes present in ≥95% of analyzed genomes (see Materials and Methods). Download FIG S2, PDF file, 0.4 MB. Copyright © 2020 Wang et al. 2020 Wang et al. This content is distributed under the terms of the Creative Commons Attribution 4.0 International license.

To further quantify the tendency of lifestyle transitions, we calculated transition rates between lifestyles using BayesTraits (see Materials and Methods). The transition rate from nodule association to nonnodule association was about eight times the rate calculated for the opposite direction (27.62 versus 3.30; log Bayes factor [logBF] = 72.04) (Fig. 2b), indicating that the tendency for rhizobia to lose their nodulating ability is much stronger than the gain of nodulating ability for nonrhizobia. The rate of transition to nodule associations was the highest for the plant-associated lifestyle (Fig. 2a) and was significantly higher than that determined for the animal-associated lifestyle (logBF = 3.28) but not that determined for the free-living lifestyle (logBF = 0.90). The transition from the plant-associated lifestyle to the free-living lifestyle is more likely to occur than the reverse transition, as reflected by the significantly higher transition rate (logBF = 3.48) (Fig. 2a). The rate of transition from an animal-associated lifestyle to any of the other three lifestyles was significantly lower than the rates of transition in the opposite direction and was not significantly different from zero (Fig. 2a). The pattern held when we combined all of the non-animal-associated lifestyles (logBF < 2; Fig. 2b). Nodule- and animal-associated bacteria exhibited markedly distinct patterns in lifestyle transition. Animal pathogens are often subjected to strong bottlenecks, which may lead to genetic drift and to massive losses of genes that cannot confer large advantages (2), and as a result, this could make it difficult for them to evolve into other lifestyles or even to reach an evolutionary end. On the other side, rhizobia need to dwell various habitats, including nodules, bulk soils, and rhizospheres; the free-living stage in soils and rhizosphere may provide rhizobia large population sizes, acting against the tendency of the decrease of selection effectiveness observed in animal pathogens (2). Rhizobia are also equipped with genes allowing them to thrive in all these habitats. Thus, they might easily shift back to nonrhizobia by loss of symbiosis genes, in particular, when their hosts grow in nitrogen-rich soils, as the benefit of carrying these symbiosis genes becomes vanishingly low (20).

### Coevolutionary history of the associations between *Rhizobiales* species and their hosts.

A large proportion (76%) of *Rhizobiales* members sampled in the present study were isolated from a host-associated environment, making it possible to explore the coevolution between *Rhizobiales* and their hosts. Our strategy started by estimating the time of origin of each lifestyle by the use of careful molecular clock analyses and comparing each estimated time of origin with that of their hosts which were recorded in fossils. For computational efficiency, we selected 176 representative genomes based on the operational taxonomic unit (OTU) at the 16S rRNA gene sequence identity level of 98.7% (21) (see Materials and Methods). Molecular clock analyses showed that the LCA of the *Rhizobiales* occurred 1,569 million years ago (Mya) (95% highest posterior density [HPD] interval, 1,667 to 1,447 Mya), which greatly predated the origin of their hosts (Fig. 3) (22–24; also see below). This lends strong support for the idea of a free-living LCA of *Rhizobiales* illustrated by the ancestral lifestyle reconstruction (Fig. 1; see also Text S1 in the supplemental material).

With an origin at 116 Mya (95% HPD interval, 146 to 88 Mya), nodulating *Bradyrhizobium* showed the earliest origin among the aforementioned four major rhizobia lineages (Fig. 3), very close to the origin time of another nodulating clade, the *Azorhizobium* clade, at 134 Mya (95% HPD interval, 180 to 91 Mya). Hence, the first alphaproteobacterial rhizobium was likely associated with *Bradyrhizobium* or *Azorhizobium*. The origins of nodulating lineages of *Mesorhizobium*, *Sinorhizobium*, and *Rhizobium* generally postdated those of *Bradyrhizobium* and *Azorhizobium* (Fig. 1), implying that the nodulating ability of the former lineages was acquired from the latter lineages or their relatives. Notably, the divergence time of the *Rhizobiales* lineages should be understood as a span of the posterior age estimate (indicated by the 95% HPD interval), rather than as a time point. Recent molecular dating studies suggest that legumes (the host of alphaproteobacterial rhizobia) originated 110 to 65 Mya (25) (see also references 16, 26, and 27 and Text S1). Hence, there were considerable overlaps in the origin times of legumes and alphaproteobacterial rhizobia (e.g., *Azorhizobium*, *Bradyrhizobium*, and *Mesorhizobium*). The origin of leguminous nodules, assuming its first appearance in the LCA of legumes (28–30), was thus roughly contemporaneous with that of nodulating alphaproteobacterial rhizobia (Fig. 3). Alternatively, certain rhizobial lineages (*Azorhizobium* or *Bradyrhizobium*) might have originated a bit earlier than legumes and evolved the nodulating ability after the emergence of legumes in independent lineages. Additionally, the uncertainties might result from methodological limits of molecular dating in organisms with ancient origins (31), such as the *Rhizobiales*. Nonetheless, our result remained compatible with the general hypothesis of the coevolution of alphaproteobacterial rhizobia and their hosts (Text S1).

The plant-associated members in *Rhizobiaceae* and *Methylobacterium* originated 280 Mya (95% HPD interval, 314 to 242 Mya) and 207 Mya (95% HPD interval, 241 to 171 Mya), respectively (Fig. 3), >100 million years (My) after the terrestrialization of plants (24). In support of this idea, these lineages are found in the microbial communities at the phyllosphere or endosphere of various land plants (8, 32). Note that, due to the lack of species that split early, the LCA shared by *Rhizobiaceae* and its sister group *Martelella* was ambiguous in terms of its lifestyle, and there is a chance that it had already adapted to a plant-associated lifestyle (Fig. 3). As this LCA occurred at 417 Mya (95% HPD interval, 479 to 355 Mya) and coincided with the emergence of land plants at ∼470 Mya, when scarce nutrients and water severely limited the development of early land plants (3), the primitive interaction between these *Rhizobiales* lineages and the earliest land plants might have contributed to the successful terrestrialization of early plants.

Animal-associated *Brucella* and *Bartonella* displayed divergent patterns in terms of the origin time (Fig. 3). *Brucella* originated no earlier than 20 Mya, whereas *Bartonella* likely originated at 343 Mya (95% HPD interval, 393 to 293 Mya), much earlier than the emergence of mammals (22), suggestive of host shifts during *Bartonella* evolution. Recent efforts have revealed that basal lineages Bartonella apis and Bartonella tamiae are nutritional commensals inhabiting the guts of insects (33) whose origin (23) generally coincided with the emergence of *Bartonella* (Fig. 3). Further, unlike *Brucella*, where transmission is directly mediated by contact between mammals, *Bartonella* is transmitted across mammals via blood-sucking arthropod vectors. The evidence provided above suggests that ancestral lineages of *Bartonella* might have already lived closely with arthropods and that they became arthropod-transmitted mammalian pathogens ∼150 My later, coinciding with the emergence of mammals (22) (Fig. 3).

To accommodate potential biases in the time constraints used here, we performed additional analyses with various combinations of time constraints derived from fossil records and estimates from previous studies (Fig. S3; see Text S1 for details). In general, the patterns obtained from these new analyses remained unchanged (Fig. S3). For example, the 95% HPD interval of the estimates for the origins of both nodulating *Bradyrhizobium* and *Azorhizobium* generally overlapped that of nodulating plants across all combinations of calibrations (Fig. S3c), strengthening the idea that the first rhizobial lineages in *Alphaproteobacteria* lay in *Bradyrhizobium* or *Azorhizobium*. In addition, note the uncertainties inherent in time estimates, which can arise from the lack of fossils from close relatives, data partitioning, and lack of gene sets (Fig. S3). In general, removal of secondary calibrations within the *Proteobacteria* (sets 5 to 8), decreasing the number of partitions (set 11), or using the single-copy genes identified by OrthoFinder (set 12) led to more-ancient posterior times being estimated (Fig. S3). The largest posterior age value was observed in Set 11, where the sequence data were partitioned according to the scheme recommended by ModelFinder instead of being fully partitioned as performed for the other 11 calibration sets (Fig. S3c). This is consistent with previous findings revealing that estimated ages increase as more partitions come to be used (34). Different lineages also showed different extents of variation in the posterior age across calibration sets (Fig. S3c). Considering these uncertainties, one should be cautious in offering any conclusive arguments based on the time estimates. We think that the lack of fossils in most major bacterial lineages, the ambiguity in the data used to estimate the age of cyanobacteria fossils, and the large phylogenetic distance between cyanobacteria and other bacteria are the biggest challenges in bacterial divergence time estimation. Future studies may consider using the recently developed strategy based on horizontal gene transfer (HGT) (35, 36) to better resolve the evolutionary timeline for *Rhizobiales* and other bacteria.

10.1128/mSystems.00438-20.4 FIG S3 Different calibrations and estimated divergence times for host associations. (a) Illustration of calibration points. (b) Time constraints used in the divergence time estimate. Set 1 was used to determine the data reported in the main text and Fig. 3. (c) HPD credible intervals (95%; vertical bar) of the time estimates of the nine origins of host-associated lifestyles for 12 different sets of time constraints (see Text S1 for details). The horizontal bar in each panel denotes the range of the times of origin of the hosts. The cartoons of legumes, mosses, mice, and bees represent legumes, land plants, mammals, and insects, respectively. Download FIG S3, PDF file, 0.3 MB. Copyright © 2020 Wang et al. 2020 Wang et al. This content is distributed under the terms of the Creative Commons Attribution 4.0 International license.

### Genome-wide identification of genes associated with nodulating members.

Gene families conserved across diverse bacterial lineages of the same lifestyle show strong signals for convergent evolution and are therefore potentially important to bacterial adaptation in their common habitats (15, 16, 37). We integrated different protein family annotations, performed BayesTraits-based analysis to search for genes significantly associated with their lifestyles using the full set of the *Rhizobiales* genomes (see Materials and Methods), and elaborated on their putative roles in lifestyle adaptation of a few examples of particular interest, starting from those correlated with nodule-associated *Rhizobiales* (Data Set S1b; see also Data Set S1c for the full list).

*Rhizobia*-legume symbiosis is initiated by nodule formation and invasion (38, 39). As expected, genes participating in the biosynthesis of Nod factors (*nod* genes), which play key roles in inducing the host plant to form infection threads (38), were among the top-ranking significantly correlated genes (Fig. 1; see also Data Set S1). Genes encoding a type III secretion system (T3SS), T4SS, and T6SS, as well as bacterial effector proteins exported through them, were also detected. Distinct from other secretion systems which simply transport proteins/compounds out of cells, T3SS, T4SS, and T6SS can transport bacterial effectors to enable them to have direct communication with the eukaryotic cytosol (40). The roles of the T4SS and T6SS in symbiosis are less well studied than those of the T3SS. Our results suggest that those secretion systems, though not universally present at the strain level, may contribute to *Rhizobia*-legume symbiosis potentially by affecting host specificity and/or nodule growth (40). Genes involved in hopanoid lipid synthesis and modification were also identified, supporting the view of hopanoid as a molecule promoting bacterial mobility and attachment to root surfaces (41). Moreover, we detected several *Rhizobia*-associated genes that may help manipulate the level of plant hormones by rhizobia, such as the 1-aminocyclopropane-1-carboxylate deaminase (*acds*) which facilitates nodulation by degrading the precursor of the nodule inhibitor ethylene (42), and halimadienyl-diphosphate synthase which presumably participates in biosynthesis of gibberellin (42).

To supply legumes with N in nodular environments, rhizobia are equipped with the nitrogenase-encoding *nif* genes, which, as expected, were identified as genes enriched in nodulating bacteria (Fig. 1; Data Set S1b). A high rate of O_2_ respiration is needed to fulfill the energy requirement during N_2_ fixation (43). However, O_2_ can irreversibly inactivate nitrogenase. The microaerobic environment of nodules sets up a conflict of interest between N_2_ fixation and other biological processes in terms of O_2_ concentration (39). To cope with this issue, some rhizobia recycle the H_2_ released from N_2_ fixation via hydrogenase to reduce energy loss by regenerating chemical energy and removing H_2_ and O_2_, the reversible and irreversible inhibitors of nitrogen fixation, respectively (44). Accordingly, the *hyd* and *hup* genes encoding this H_2_ recycling system were significantly associated with nodule-associated taxa. In addition, many O_2_-high-affinity cytochromes and genes involved in their synthesis or regulation were significantly correlated. The best example is the Fix system (Fig. 1), including *fixABCX* participating in electron transfer to nitrogenase, *fixJLKT* responsible for *nif* regulation, and *fixNOQP* encoding high-affinity cytochrome oxidases, which collectively ensures high efficiency of aerobic biological processes under micro-oxic environment (39). Also significantly correlated were denitrifying enzymes, which are hypothesized to participate in detoxification of the signaling module nitric oxide in *Bradyrhizobium* and *Sinorhizobium* (45). In bacteroids, N_2_ fixation is driven by oxidation of host-supplied C4-dicarboxylic acids (43). Accordingly, the *dct* genes encoding the C4-dicarboxylate transporter were enriched in rhizobia. Note, however, that the low O_2_ level in bacteroids may inhibit the TCA cycle due to imbalanced redox state of NADH/NAD^+^, thereby disrupting N_2_ fixation (43). Rhizobia use the storage of polyhydroxybutyrate (PHB), which could account for half of the dry weight of rhizobial cells, as a way to stabilize cellular redox conditions, deposit redundant energy, and release the inhibition of the TCA cycle at low O_2_ conditions (43). Accordingly, several genes (e.g., *phaZ*, *phbB*, and *phbC*) responsible for the synthesis and degradation of PHB were found to be significantly associated.

Rhizobia need to not only persist in nodules but also thrive in soils where they compete with other bacteria for limited resources such as iron (Fe) and phosphorus (P). To overcome the low concentrations of soluble Fe available in soils, rhizobia use siderophores to import Fe by binding it in tight complexes (46). Indeed, several genes involved in siderophore biosynthesis and transport were significantly correlated with nodule-associated bacteria. Likewise, genes comprising the *phn* operon encoding a C-P lyase for utilization of phosphonate, which is commonly found in soils (47), were also among *Rhizobia*-associated genes. In addition, several genes involved in the biosynthesis of ectoine, a protectant against osmotic stress described in many soil bacteria (48), were significantly correlated with rhizobia, implying their role in adaptation to distinct osmotic pressures across various habitats.

### Genes associated with animal-associated, plant-associated, and free-living lifestyles.

Using the same procedure, we identified genes significantly correlated with each of the other three lifestyles. Fewer genes were significantly correlated with animal-associated than with nodulating bacteria (Data Set S1d), likely because of the limited taxonomic distribution of the animal-associated lifestyle within the *Rhizobiales*. Of most interest were the VirB genes encoding the type IV secretion system (Fig. 1), known as the essential virulence factors contributing to the success of infection by *Bartonella* and *Brucella* (49). Also significantly correlated with the animal-associated bacteria were the genes encoding adhesins, which are necessary for attaching *Bartonella* to host cells (49). Likewise, many genes that encode transporters of amino acids and metals (e.g., Fe^3+^ and Mg^2+^) showed significant correlation, consistent with their demonstrated essentiality in the survival of *Bartonella* and *Brucella* in mammalian bloodstream (50, 51) (Data Set S1b). Genes coding for multidrug efflux systems, which may help pathogens develop antimicrobial resistance during infection (52), were also enriched in animal-associated bacteria (Data Set S1b).

In addition, genes encoding the class Ib ribonucleotide reductase (RNR), a key enzyme in synthesis of the precursors of DNA in its replication (53), were among significantly associated genes in animal pathogens. Compared with other types of RNRs, class Ib RNR is special because it uses manganese instead of iron as the cofactor and is found only in bacteria and bacteriophages (53). The use of class Ib RNR in DNA replication thus likely helps *Bartonella* and *Brucella* survive in mammalian cells where iron is rare and facilitates escape from host defense via iron limitation, making the class Ib RNR a promising target for antibacterial and antiviral drug design, particularly given its absence in eukaryotes (thereby exerting less influence on host cells) (54).

A considerable number of genes significantly correlated with plant-associated strains encoded transporters covering diverse substrates, including sugars, amino acids, and oligopeptides (Data Set S1e). Many of them participate in the transport of rhamnose, a key component of lipopolysaccharide important to bacterial attachment to plants (55). Also included were those involved in cellulose synthesis and its activation, consistent with their roles in mediating plant-bacterium interaction (56). Free-living *Rhizobiales* species were derived from diverse isolation sites (Data Set S1). Hence, there were few “characteristic” genes shared by free-living *Rhizobiales* (Data Set S1f). Of most interest was the *arc3* gene encoding a protein pumping arsenite from the cell, which showed the lowest *P* value among all genes correlated with this lifestyle, suggesting its role in adaptation to arsenite compounds, the most prevalent environmental toxic substances (57).

### Genome expansion in the origin of nodulating lineages.

Lifestyle-associated genes could be acquired before (i.e., preexisting traits), during, or after a lifestyle transition. These scenarios were differentiated using ancestral genome reconstruction of 176 representative *Rhizobiales* genomes with BadiRate (see Materials and Methods). Overall, this analysis predicted that *Rhizobiales* started with ∼2,100 genes at its LCA and gradually expanded to the current genome sizes (Fig. 4a). It also predicted repeated genome expansion based on the origin of the nodule-associated lifestyle and repeated genome reduction based on the origin of the animal-associated lifestyle (Fig. 4a), consistent with an early study that analyzed only nine genomes (12).

The *nod* and *nif* genes appeared to be the only ones that were repeatedly gained during the lifestyle transitions to the four major nodulating lineages, possibly via HGT from rhizobial lineages that had evolved early (14), consistent with their determining role in nodulation and nitrogen fixation by rhizobia. In contrast, genes constituting the Fix system, Dct transporter system, and *phn* operon and those involved in PHB metabolism were already present in their nonnodulating ancestors (Fig. 4b).

The evolutionary histories of most *Rhizobia*-associated genes were actually a mix of these scenarios where they preexisted in some rhizobia but were not recruited until lifestyle transition in others. Examples are T3SS, T4SS, T6SS, and denitrification genes (Fig. 4b). Such distinct patterns across lineages were closely related to the ancestral lifestyle from which the corresponding rhizobial lineage evolved, implying that these genes contributed to lifestyle adaptation in a lineage-specific manner (13). For instance, the nodulating *Bradyrhizobium* species likely arose from a free-living ancestor (Fig. 1). Accordingly, most genes involved in bacterium-host interactions, such as T3SS, T4SS, and T6SS genes, were predicted to be absent in the nonnodulating ancestor of *Bradyrhizobium* (Fig. 4b). T3SS was inferred to be acquired by the ancestor of nodulating *Bradyrhizobium*. This is interesting because photosynthetic *Bradyrhizobium* lineages that split early can use T3SS to nodulate legumes in the absence of *nod* (58). Considering its reported roles in symbiosis and conservation in *Bradyrhizobium* (40), our results imply that a gain of T3SS might have contributed to the ancestral transition of *Bradyrhizobium* from the free-living to the nodulating lifestyle (Fig. 4b). Distinct from *Bradyrhizobium*, nodulating *Rhizobium* and *Sinorhizobium* likely evolved from a plant-associated ancestor (8) (Fig. 1). Plant-associated bacteria produce various cell surface polysaccharides, among which EPS (exopolysaccharide) attracts the most attention because of its high abundance in the surrounding environment of bacteria (59). EPS has been shown to contribute to both symbiosis for *Rhizobium* and *Sinorhizobium* and plant pathogenesis for *Agrobacterium* (60). Our results suggest the presence of EPS I (succinoglycan) synthesis genes in the LCA of *Rhizobiaceae* and in the shared ancestors of *Rhizobium*, *Sinorhizobium*, and *Agrobacterium* (Fig. 4b). We hypothesize that the role of EPS in early *Rhizobiaceae* lineages may be simply the attachment to the plant surface and a protective barrier (as in the case of *Agrobacterium*) (60) and that it was coopted later in evolution by some rhizobia as a signaling molecule during invasion and infection thread formation. Note that, in addition to EPS I, rhizobia secrete other types of EPSs. However, given their low abundance and that their synthesis genes are less well characterized in rhizobia (59), those genes were not analyzed here. The case for *Mesorhizobium* was similar to those for *Rhizobium* and *Sinorhizobium*, where the genes participating in EPS synthesis and T6SS were inherited from their nonnodulating ancestor (Fig. 4b). This implies a plant-associated ancestor of *Mesorhizobium*, although the ancestral lifestyle of *Mesorhizobium* could not be fully resolved (Fig. 1). The conservation of T6SS in nodulating *Mesorhizobium* hints at its role in symbiosis, despite there being little knowledge about it (40). Further, we employed AnGST, a method fundamentally different from BadiRate (see Materials and Methods), to repeat the ancestral reconstruction analysis and obtained a similar pattern (Fig. S4).

10.1128/mSystems.00438-20.5 FIG S4 The presence/absence of lifestyle-associated genes in extant ancestral nodes (solid/white circles) and key ancestral nodes (solid/white squares; inferred by AnGST) of major nodule-associated lineages (a) and animal-associated lineages (b). Download FIG S4, PDF file, 0.5 MB. Copyright © 2020 Wang et al. 2020 Wang et al. This content is distributed under the terms of the Creative Commons Attribution 4.0 International license.

Despite significant genome expansion in the origins of nodulating lineages (Fig. 4a), genes encoding the gene transfer agent (GTA), a small tailed phage-like element that consists of ∼15 genes and mediates HGT of short genomic DNA segments (61), were possibly independently lost in all of the four major nodulating lineages (Fig. S5). A previous study showed that, while GTA was retained in *Azorhizobium*, its expression is suppressed when members of this rhizobial lineage form bacteroids (62). Also, despite the importance of HGT via plasmids or mobile islands to the *Rhizobia*-legume symbiosis (12), GTAs may not play a role in this process, because they preferentially mediate transfer of chromosomal DNA (63, 64) and can pack DNA segments of ∼4 kb (61), which are much smaller than those needed for rhizobial symbiosis. It is likely that GTAs became dispensable and thus were lost during the evolution toward the rhizobial lineages. This hypothesis can be tested by introducing the full set of GTA genes into rhizobia and assessing the effects on the growth of the bacteria.

10.1128/mSystems.00438-20.6 FIG S5 The phyletic pattern of genes encoding the gene transfer agent (GTA) in the *Rhizobiales*. The first layer surrounding the phylogeny denotes the lifestyle, in which red, green, orange, and blue represent nodule-associated, plant-associated, animal-associated, and free-living lifestyles, respectively (as described for Fig. 1). The next two layers represent the presence/absence of genes encoding RcGTA (green), a widely distributed GTA first discovered in Rhodobacter capsulatus (90, 91), and BaGTA (red), which is specific to *Bartonella* (92), respectively. Homologs of RcGTA and BaGTA were identified by BLASTP using reference proteins as queries with the cutoff E value 1E−3 (90). Reference sequences of RcGTA and BaGTA were obtained from references 90 and 93, respectively. Download FIG S5, PDF file, 0.4 MB. Copyright © 2020 Wang et al. 2020 Wang et al. This content is distributed under the terms of the Creative Commons Attribution 4.0 International license.

Exchange of genetic materials by HGT between bacteria inhabiting the same niches is very frequent (65). Given the coexistence of nonnodulating bacteria with rhizobia in both nodular and soil environments, it is likely that key genes involved in rhizobial symbiosis (e.g., the *nod*, *nif*, and *fix* genes) have frequently been transferred across bacteria over time (14). Paradoxically, until now, the hundreds of known rhizobial species were discovered in some 12 genera within only the *Rhizobiales* and *Burkholderiales* (14, 66). This suggests that HGT of these symbiosis genes is insufficient for the conversion from nonrhizobia to rhizobia. A recent study revealed that genome modifications of the recipient lineages are required for newly acquired symbiosis traits to function (67). In the present study, we found that preexisting traits and gene losses might have also contributed to the origin of rhizobia. Possibly, it is a combination of different mechanisms that make diverse lineages within the *Rhizobiales* independently evolve to be successful symbionts of legumes, which, however, needs to be further explored.

### Genome reduction and gene gain/losses in animal pathogens.

The origins of animal pathogens were characterized by significant genome reductions (Fig. 4a). In particular, many genes involved in amino acid metabolism were lost during the evolution of *Bartonella*, leading to the incompleteness of most amino acid biosynthesis pathways in this lineage (33). To compensate for these losses, *Bartonella* recruited several amino acid uptake genes during and after the lifestyle transition to make use of the available resources in the host (Fig. 4c; see also Fig. S4b). In contrast, most genes gained by *Brucella* are involved in the uptake of metals, including nickel and cobalt (Fig. 4c; see also Fig. S4b). These genes have been proven critical to the virulence of other Gram-negative bacteria (reviewed in reference 68) and might be similarly important to *Brucella* (18). Other genes, e.g., those encoding T4SS, class Ib RNR (ribonucleotide reductase), and multidrug exporters, were likely present at the LCA shared by *Bartonella* and *Brucella* (Fig. 4c; see also Fig. S4b). These genes might make related lineages “preadapted” to animal association and pathogenesis. Of note, some strains belonging to *Ochrobactrum*, representing the closest relatives of *Brucella*, have evolved as opportunistic pathogens. People with an indwelling medical device are most susceptible to the bloodstream infection by *Ochrobactrum* (69). This could result from its ability to resist antibiotics and adhere to synthetic materials (70), consistent with the idea that the LCA of *Ochrobactrum* and *Brucella* might have already harbored relevant genes (Fig. 4c; see also Fig. S4b). We speculate that these preexisting genes might have been involved in different but related pathways in the free-living ancestors and might evolved new functions in pathogenesis during evolutionary transition to the host-associated lifestyle. For example, the T4SS in *Bartonella*, which exclusively functions to modulate the bacterium-eukaryotic host cell interaction, likely evolved from an ancestral conjugation system involved in the formation of stable mating junctions (71). The findings described above also suggest that to limit the risk of emergence of pathogenic bacteria, it is important to watch for (i) those with genes that potentially facilitate adaptation or conversion to a pathogenic lifestyle in genomes and (ii) those that infect related hosts or that have already been adapted to the host (72). Such examples include the aforementioned *Ochrobactrum*. In addition, several members of the *Afipia* are associated with the blood of patients (Data Set S1), highlighting its potential as a reservoir for emerging diseases (73).

### Caveats and concluding remarks.

Like a recent study (37), we classified host-associated *Rhizobiales* based on their isolation sites. However, different lifestyles could represent dynamic alternatives available to the organism at different time points (7). In our data sets, three non-nodule-associated strains (*Mesorhizobium* sp. strain LCM 4577, Rhizobium phaseoli Ch24-10, and *Rhizobium* sp. strain NXC14) possess a complete set of *nodABC* genes, thus likely representing strains that are capable of nodulating legumes but that happened to be isolated elsewhere. Thirteen of the 244 nodule-associated strains do not carry any *nodABC* genes. While some of them belong to the photosynthetic *Bradyrhizobium* lineage which utilizes a Nod-independent strategy for nodulation (58), others may represent nonnodulating bacteria living in nodules. Moreover, although our computational identification of lifestyle-correlated genes provides useful insights into the development of microbe-based strategies for sustainable agriculture and for prevention of plant/animal diseases, it is based purely on bioinformatics analyses. The detailed functions of many of identified genes still await exploration. The overrepresentation of agriculturally and medically important strains in sequenced genomes (8), although accounted for by BayesTraits, should also be noted.

Given the prevalence of plant-associated members, a recent study hypothesized that the LCA of *Rhizobiales* was already capable of colonizing the root of plants (8). Here, we revisited this idea by showing that the highly diversified lifestyles in *Rhizobiales* likely originated from a free-living ancestor. We also estimated the emergence of *Rhizobiales* some 1,500 Mya, far predating the origins of their hosts, including land plants (∼470 Mya) (24), insects (∼480 Mya), (23) and mammals (∼180 Mya) (22). Such an ancient origin of *Rhizobiales* indicates that the free-living lifestyle was adopted by *Rhizobiales* over the first half of their evolutionary trajectory. *Rhizobiales* evolved to live in association with early terrestrial animals and plants that emerged during the Cambrian explosion in their middle age, and at a later stage, when nodulating plants emerged, they established the legume-rhizobium symbiosis which is among the best-known symbiosis relationships.

Our results also indicate that genomic plasticity is an important feature driving the evolution of diverse lifestyles in *Rhizobiales*. Both the recurrent gains of genes that determine lifestyle transition and the losses of genes that do not have big advantages underlie the evolutionary adaptations of *Rhizobiales* to different lifestyles. Some of these lifestyle-important genes, if acquired by ancestral lineages, might make their descendants preadapted to the development of certain lifestyles. In addition, HGTs of genes important for and common to *Rhizobiales* of different lifestyles (e.g., T4SS genes) across rhizobia, plant associates, and animal pathogens might facilitate lifestyle diversification across the entire order (6, 13).

## MATERIALS AND METHODS

### Collection of *Rhizobiales* genome sequences and lifestyles.

We retrieved genome sequences and annotations of 1,264 *Rhizobiales* isolates (i.e., no metagenome-assembled genome was included) from NCBI RefSeq (last accessed July 2017), estimated the completeness of each genome using CheckM v1.0.7 with the default parameters (74), and collected their lifestyle information from the BioSample and BioProject database at NCBI and from literature (see Data Set S1 in the supplemental material for the complete information). Those with a completeness value lower than 99% or without a clearly documented lifestyle information were removed. We selected these stringent criteria because genomes with low completeness may confound gene gain/loss analysis. Genomes without an identified 16S rRNA gene were also excluded. The remaining 891 strains were classified into four lifestyles based on their isolation site (37). Strains isolated from nodules are referred to as nodule associated. Those isolated from other parts of plants, the rhizosphere included, were considered plant-associated strains (37). Those isolated from animals were defined as animal-associated strains. Bacteria that were not associated with any host were classified as free-living strains; among those strains, 31%, 43%, and 12% were isolated from bulk soil, freshwater, and the ocean, respectively.

### Phylogenomic tree construction.

Amino acid sequences of 53 conserved ribosomal protein genes, which are less affected by HGT and thereby generally reflect well the evolutionary relationships of the genomes carrying them (75), were retrieved based on the results of reversed PSI-BLAST (https://www.ncbi.nlm.nih.gov/Structure/cdd/wrpsb.cgi). Sequences were aligned using MAFFT v7.222 with default parameters (76). Alignments were trimmed using trimAL v1.4.1 (77) with the parameter “-st 0.001” and were concatenated for phylogenetic analysis. For genomes with identical ribosomal protein sequences, one genome was randomly chosen as the representative, which led to retrieval of a total of 655 genomes (Data Set S1) for subsequent analyses.

The phylogenomic tree of *Rhizobiales* was built using IQ-Tree v1.5.3 (78) with the parameter “-s alignment -spp partition -m MFP+MERGE -rcluster 10 -mset LG,WAG,JTT -mrate R -wbtl -bb 1000” and with six genomes from other *Alphaproteobacteria* lineages (Caulobacter crescentus CB15, Rhodobacter sphaeroides 2.4.1, Leisingera daeponensis DSM 23529, Sphingomonas melonis FR1, Porphyrobacter cryptus DSM 12079, and Rhodospirillum rubrum F11) used as the outgroup. This procedure automatically implements a data partition model for the concatenated alignment and determines the best-fit substitution model for each partition. A total of 1,000 ultrafast bootstrap approximations (79) were performed to get the support value for each node. Those with a support value of ≥95% were considered to be well-supported nodes (79). Phylogenies were visualized with iTOL v4 (https://itol.embl.de/).

### Calculation of rates of transition between lifestyles and reconstruction of ancestral lifestyles.

Rates of transition between lifestyles were calculated using the reversible-jump Markov chain Monte Carlo (MCMC) method implemented in BayesTraits v3.0.1 (19), which takes into account both the topology and the branch length of the species phylogeny and is widely used in comparative phylogenetic studies. The analysis was performed across the 1,000 ultrafast bootstrap trees generated by IQ-Tree and was run for 10,000,000 iterations (1,000 stones, each sampled for 10,000 iterations) after discarding the first 1,000,000 runs as representing the burn-in. All priors were set to an exponential with a mean of 10 (80). Convergence was checked using Tracer v1.6 (http://tree.bio.ed.ac.uk/software/tracer/). Alternative hypotheses were tested using log Bayes factor (logBF) calculated as 2 × (log marginal likelihood [model I] – log marginal likelihood [model II]) (19). Values of logBF between 2.0 and 5.0, between 5.0 and 10.0, and above 10.0 were considered positive evidence, strong evidence, and very strong evidence for support, respectively (19).

Ancestral lifestyles were inferred using the parsimony model in Mesquite v2.7.5 (https://www.mesquiteproject.org/), which finds the ancestral lifestyles that minimize the number of steps of lifestyle changes based on the species phylogeny and observed lifestyle distribution, as used in many studies (5, 15). We further validated major origins of host-associated lifestyles (i.e., the nodule-, plant-, and animal-associated lifestyles) with the maximum likelihood method using the MultiState module implemented in BayesTraits. To accommodate the fast transitions across short branches, which could lead to overestimations of transition rates and thus to inaccurate ancestral state reconstruction, we estimated the kappa parameter to be 0.076 in BayesTraits, which maximizes the likelihood of the inference (see Fig. S2a in the supplemental material). Ancestral lifestyles were also inferred based on the genome tree built with a complete taxon sampling (i.e., without removing any genomes with low completeness) (Fig. S2b) and on phylogenies constructed by the use of 101 single-copy genes identified by OrthoFinder v2 (81), allowing a gene family member to be absent in at most 5% genomes (Fig. S2c).

### Estimating the evolutionary timeline of the *Rhizobiales*.

For computational efficiency, we classified all *Rhizobiales* genomes into 171 OTUs at the 16S rRNA gene identity level of 98.7%. For host-associated lineages of *Brucella*, *Rhizobium*, and *Sinorhizobium*, we applied a cutoff of 16S rRNA identity at 99.5% (82) to increase the number of the OTUs of these lineages, as using a cutoff at 98.7% (21) can capture only two OTUs of each of those lineages and may lead to inaccurate time estimations. For each OTU, a reference strain’s genome was selected if available. Otherwise, one genome was randomly chosen (see Data Set S1 for the list). Further, to better constrain the origin times of nodulating lineages in *Bradyrhizobium*, we included *Bradyrhizobium* sp. strain URHD0069 and *Bradyrhizobium* sp. strain URHA0002, the two basal lineages of *Bradyrhizobium* adapting to a free-living lifestyle (Fig. 1). Selected genomes from *Alpha-*, *Beta-*, and *Gammaproteobacteria* were also included as additional outgroups (11).

The amino acid sequences of 25 universally conserved single-copy genes were chosen as characters for molecular dating, as used in a previous study (9). Their amino acid sequences were aligned using MAFFT v7.222 (76) and trimmed using TrimAl v1.4.1 (77). All alignments were manually checked to avoid poorly aligned sequences. The relaxed molecular clock analysis was performed using MCMCTree from PAML v4.9i (83), a widely used MCMC-based tool for molecular dating (31). Since the use of more partitions can improve the precision of divergence time estimates (34), each gene was allowed to have its own partition, resulting in a total of 25 partitions in MCMCTree analysis. We used the approximate likelihood calculation and independent rate model implemented in MCMCTree. The first 10,000 iterations of each MCMC chain were discarded as burn-in, and the chain was run for 50,000 iterations, sampling every two iterations. Convergence was checked by repeating the analyses described above (see “Data availability” below).

MCMCTree employs soft fossil constraints for each calibration point, allowing 2.5% of the posterior probability distribution to exceed the minimum or maximum ages specified by the user (83). For the majority of the calibration points, the priors of the minimum and maximum ages were based on fossil and geologic evidence from cyanobacteria (Fig. S3; see also Text S1 in the supplemental material), as such evidence is most abundant in cyanobacteria among bacteria (84). In addition, given the potential for inaccuracy due to the use of very distantly related lineages as calibration points (31), we included two calibration points within the *Proteobacteria* based on other literature (see Fig. S3a for the full list of calibration points and Text S1 for the justification for each calibration point). To check whether the results were biased by the selected time constraints, we performed the same analysis with other different combinations of time constraints based upon other studies (Fig. S3).

### Functional annotation of gene families.

Clustering of genes and protein domains basically followed a recent study (37). For each genome, we clustered proteins into families based upon the annotation resources of COG, TIGRFAM, and KEGG Orthology. E values derived from reverse PSI-BLAST that were equal to or lower than 1e−5 were considered to represent homologs. HMMER v3.1 (85) was used to perform domain annotation for each gene based on Pfam release 30.0 with the gathering threshold and an E value cutoff of 1e−5. Furthermore, to capture the remaining genes that might not have functional annotations in the databases mentioned above, OrthoFinder v2 (81) was used with a cutoff of 1e−10 to cluster protein sequences from all analyzed genomes into homology-based orthogroups, which were used to infer the ancestral genome content (Fig. 4a).

### Identification of lifestyle-associated genes.

Genes significantly associated with lifestyles were identified using BayesTraits v3.0.1 (19). BayesTraits takes a binary matrix composed of two traits (e.g., nodule-associated lifestyle versus non-nodule-associated lifestyle) and determines whether the presence/absence of a gene family and the change in the lifestyle occurred dependently. It allows the identification of genomic adaptations that are associated with parallel switches in lifestyles. We applied the likelihood ratio test (LRT) to test the evolutionary association between the gene family and a lifestyle. In brief, we calculated the likelihood ratio associated with each gene family as representing twice the difference between the log likelihoods of the two models (2ΔlnL, where ΔlnL = lnL_independent_ – lnL_dependent_) against a χ^2^ distribution with four degrees of freedom (15, 16, 86). To determine the direction of association (i.e., whether the lifestyle is correlated with the presence or absence of the gene), we calculated the transition rate difference as ΔQ = q21 + q31 + q34 + q24 − (q12 + q13 + q43 + q42) (86). As shown in Fig. S6, we would expect a positive correlation between the lifestyle and the presence of the gene (i.e., we would expect the shaded regions to dominate) for ΔQ values of >0 (86). We identified genes as positively associated with a lifestyle if their false-discovery-rate (FDR)-adjusted (Bonferroni method) *P* values derived from the LRT were lower than 0.05 and ΔQ values were above zero. It is worth noting that the same gene could be phylogenetically correlated with bacteria of two or more lifestyles if it meets the criteria described above.

10.1128/mSystems.00438-20.7 FIG S6 Illustration of possible combinations of two binary characters (gene and lifestyle) and of the transition rates (qXX) between these combinations. The shaded combinations dominate when the gene is positively correlated with the lifestyle (in this example, nodule association) calculated by BayesTraits. For example, when the q24 value is greater than the q42 value, that means that in the presence of the gene, the conversion from a non-nodule-associated lifestyle to a nodule-associated lifestyle occurs more easily than the opposite process. When the q31value is greater than the q13 value, that means that in the absence of the gene, it is easier for nodule-associated bacteria to lose a nodule association than for non-nodule-associated bacteria to become nodule associated. The rate difference is calculated as follows: ΔQ = q21 + q31 + q34 + q24 − (q12 + q13 + q43 + q42). Significant positive correlation between the gene and the trait should meet the following two criteria: (i) FDR-corrected *P* values (Bonferroni correction) should be <0.05, and (ii) ΔQ values should be >0. Download FIG S6, PDF file, 0.01 MB. Copyright © 2020 Wang et al. 2020 Wang et al. This content is distributed under the terms of the Creative Commons Attribution 4.0 International license.

### Ancestral genome reconstruction and inference of gene gains and losses.

Evolution of genome content via gene gains and losses was reconstructed using BadiRate v1.35 (87) with the parameter “–ep CSP –anc -rmodel BDI -bmodel FR.” By employing the Sankoff parsimony method, this parameter estimates lineage-specific rates of gains and losses for each gene family while considering both the topology and branch lengths of the phylogeny (87).

In addition, we applied AnGST (88) to examine whether the general pattern shown by the BadiRate analysis was robust. AnGST differs from BadiRate in that it infers gene gains and losses based on reconciliation of topological incongruences between gene trees and species trees. Additionally, AnGST accounts for uncertainties in gene trees by incorporating the first 100 bootstrap trees generated by the IQ-Tree ultrafast bootstrap approximation. It also allows users to specify the penalty scores of HGT, gene duplication, and gene loss, which were determined to be 3, 2, and 1, respectively, by minimizing genome size flux as suggested by a previous study (88). Gene families with fewer than four members were excluded from AnGST analysis, as IQ-Tree cannot build trees for them.

### Data availability.

The original sequences, phylogenetic trees, molecular dating analyses, and gene gain/loss results, together with the codes generating them (89), are available at https://figshare.com/articles/Evolutionary_timeline_and_genomic_plasticity_underlying_the_lifestyle_diversity_in_Rhizobiales/9849539.

10.1128/mSystems.00438-20.8 FIG S7 Gene phylogenies of symbiosis genes built with concatenated alignment of *nodABCIJ* using IQ-Tree. Representative genomes from each *Rhizobiales* OTU used in molecular dating analysis together with rhizobia from *Betaproteobacteria* included in a recent study (14) and four *Actinomycetes* genomes carrying the *nod* genes (94) were used in gene tree construction. The trees are rooted based on midpoint rooting (where the root is put at the midpoint of the longest path between any two tips). The support values obtained from the ultrafast bootstrapping implemented in IQ-Tree are shown next to the corresponding nodes. Branches with support values of less than 80% are condensed, resulting in polytomy. Download FIG S7, PDF file, 0.05 MB. Copyright © 2020 Wang et al. 2020 Wang et al. This content is distributed under the terms of the Creative Commons Attribution 4.0 International license.

10.1128/mSystems.00438-20.9 FIG S8 Venn graph illustrating the overlap of identified genes significantly correlated with nodule associations between the representative genome and the full set of genomes. The following two methods were applied to adjust the *P* values to correct the inflation of type I errors in multiple comparisons for the representative genome set: the Bonferroni method (95), which is very conservative, and the Benjamini-Hochberg (BH) method (96), which is more relaxed. The results are compared with those identified using the full genome set (Bonferroni correction). Those with a rate difference above zero and an adjusted *P* value lower than 0.05 are displayed (see also Materials and Methods). The correlated genes for the full and representative genomes are available in Data Set S1c and at https://figshare.com/articles/Evolutionary_timeline_and_genomic_plasticity_underlying_the_lifestyle_diversity_in_Rhizobiales/9849539, respectively. Download FIG S8, PDF file, 0.02 MB. Copyright © 2020 Wang et al. 2020 Wang et al. This content is distributed under the terms of the Creative Commons Attribution 4.0 International license.
