# Supplementary material for: Evolutionary Timeline and Genomic Plasticity Underlying the Lifestyle Diversity in Rhizobiales
Source: mSystems. 2020 Jul 14;5(4):e00438-20. doi: 10.1128/mSystems.00438-20 (PMC7363004; doi:10.1128/mSystems.00438-20)
Supplement: TEXT S1 [file mSystems.00438-20-s0001.docx]

**Part A. Supplementary Discussion**

**A free-living LCA of *Rhizobiales***

Both ancestral reconstruction (Fig. 1) and molecular dating analysis (Fig. 3) point to a free-living LCA of *Rhizobiales*. However, there are reports of associations between *Rhizobiales* and eukaryotic algae or fungi [reviewed in (1)], which originated earlier than land plants and animals, hinting that there is a possibility that ancestral *Rhizobiales* have developed the basic ability to interact with eukaryotic microbes. Nevertheless, this possibility might be relatively low given the few (6/655) phycosphere-associated isolates sampled in the present study (Data Set S1) and that most algae-interacting *Rhizobiales* are late-branching lineages like *Rhizobium* and *Mesorhizobium* (1), though future research is needed to explore this hypothesis.

**The evolutionary timeline of *Rhizobiales***

Our work provides a timeline of the evolution of the lifestyle diversity of *Rhizobiales*. It is important to mention the difficulties and thus uncertainties in reconstructing the evolutionary timeline of species having diverged for such a long time. A pioneering work calculated the divergence time of *Bradyrhizobium*, *Mesorhizobium*, *Rhizobium* and *Sinorhizobium* to be 553-507 Mya (2), much more recent than the estimated time in the present study (1131 Mya [95% HPD interval 1218–1055 Mya]). Turner and Young 2000 (2) based their analysis on i) three fossils from animals and plants, very distantly related to rhizobia, ii) sequence of a duplicate gene (glutamine synthetase), and iii) the assumption of a global substitution rate across all lineages. All of these lead to biases in time estimates that are difficult to deal with (3), particular in early years when advanced algorithms had not been developed. Possibly due to these biases, the time estimate of that study (2) differs a lot from those estimated in the present study and other studies (1236-1042 Mya) (4–6).

A co-evolutionary timeline can be constructed by comparing the origin time of host-associated *Rhizobiales* and their hosts (Fig. 3). However, there are debates on the origin time of the eukaryotic hosts of *Rhizobiales*. For example, some studies suggested that the legume family (*Fabaceae*, order *Fabales*) originated 65-60 Mya (7), more recent than an estimate of 110-65 Mya referenced in the present study (8–11). We speculate that the difference is (in part) a consequence of the maximum age constraints used in the dating analysis in different studies. Of note, some studies assigned a fixed age of 65-60 Mya as the crown (7) or total group (12) of the legumes. Apparently, by imposing such a maximum constraint, these studies precluded any possibility that legumes originated before that time. Fossils, by themselves, only provide the minimum bound of certain lineages, therefore attention should be paid when specifying the maximum bound (13). In an early study (7), Lavin et al., 2015 fixed the age of the total group of legumes as 60 Mya according to (Magallón and Sanderson, 2001) (14), which, however, plausibly results from misinterpretation of the latter study where the 60 Mya was actually used as the minimum bound based on fossil record (15). Further, when Lavin et al. successively moved back the fixed root age from 60 Mya to 70 Mya, the age of the crown group of legumes increased linearly from 59 Mya to 68 Mya (7). Consequently, recent works estimated an much older origin of legumes when the putatively arbitrarily assigned maximum or fixed age constraints are removed (8–11). All the above evidence implies that the origin of legumes is underestimated in early studies. Perhaps, a more accurate estimate of the co-evolution timeline needs more high-quality fossils and a better taxonomic sampling to calibrate. Similar issues also exist for insects (16, 17), the presumable hosts of the early lineages of *Bartonella*.

In addition to *Rhizobiales*, rhizobia have been found in *Burkholderia* and *Cupriavidus* (β-rhizobia) (18, 19). Moreover, species from the actinobacteria *Frankia* can initiate the formation of nodules with some 24 plant genera distributed in *Cucurbitales*, *Fagales* and *Rosales* (actinorhizal plants) (20, 21). Thus, an interesting question would be, which lineage did the first nodulating bacteria arise from? Recent studies proposed a single-gain/massive-loss model where nodulation appeared in the LCA of nodulating plants (*Fabales*, *Cucurbitales*, *Fagales*, and *Rosales*) followed by extensive losses of this trait in most decedents (22–24). In this view, given the wider taxonomic distribution of the plants that are nodulated, early nodulating plants are more likely nodulated by *Frankia*, and later in evolution, α-/β-rhizobia nodulated the LCA of legumes presumably after acquiring keys genes by HGT. Further, phylogenetic analysis of *nodIJ* implies that β-rhizobia originated before α-rhizobia (25). This result, though inspiring, is not convincing because in their analysis the authors excluded *Azorhizobium*, which happened to be the most ancient α-rhizobia (Fig. 3) and which have the most divergent sequences of *nod* among all rhizobia (Fig. S7) (26, 27). To gain more insights into this issue, it would be helpful to calculate the divergence time of β-rhizobia and *Frankia*, and compare it with α-rhizobia. This, however, is beyond the scope of the present study and may await future research to investigate.

**The identification of lifestyle-correlated genes**

Lifestyle-correlated genes were identified on top of the phylogenomic tree built with the full list of 655 genomes, which contains many short branches representing closely related strains, which might leading to inflated transition rates and an arbitrary large number of lifestyle-correlated genes if many genes are frequently gained and lost across extremely short branches (28). We therefore performed the same analysis to identify rhizobia-correlated genes with the 176 representative genomes, where very short branches are rare (Fig. 3). As expected, fewer correlated genes were identified (Fig. S8). Nevertheless, the vast majority of rhizobia-correlated genes identified by using the representative genome set were also identified by using the full genome set (Fig. S8). This, together with the above functional interpretation of identified rhizobia-correlated genes, suggests that the identification of rhizobia-correlated genes (Data Set S1) is in general robust to the influence of short branches in the full tree.

**Part B. Selection of genes included in Figure 1 and Figure 4.**

1. Nod genes: The common *nod* genes in most rhizobia are often represented as *nodABCIJ* (29, 30). *nodABC* are responsible for the synthesis of the core of the Nod factors (31), and *nodIJ* are involved in the secretion of nod factors (32).
2. T3SS, T4SS, and T6SS: Key genes of the three secretion systems were selected according to the database EffectiveDB (33). *virB1*, *virB5*, and *virB7* were added for T4SS as they are not included in EffectiveDB but are important components of T4SS (34).
3. Hopanoid synthesis genes: The product of *shc* (squalene-hopene cyclase) is the key enzyme in hopanoid synthesis by catalyzing the conversion from 2,3-oxidosqualene squalene to hopanoid (35).
4. Plant hormone synthesis: In *Mesorhizobium loti*, *acdS* encodes a 1-aminocyclopropane-1-carboxylic acid (ACC) deaminase during symbiosis with the host legume, which catalyzes the conversion of ACC to 2-oxobutanoate. ACC is a precursor to ethylene, an important negative plant growth regulator. Thus, ACC deaminase can reduce the emission of ethylene from the legume, thereby promoting the growth of the host plant (36, 37). The halimadienyl-diphosphate synthase (K17811) is likely involved in the synthesis of gibberellin, an important hormone that promotes plant growth (38). These two genes are top-ranking ones in the identified rhizobia-associated genes.
5. EPS I synthesis genes: Most EPS I synthesis genes were not identified to be significantly correlated with nodule-associated isolates. However, given that many studies report their roles in the establishment of successful symbiosis in some rhizobial lineages, *Sinorhizobium* in particular (39, 40), these genes were included. Shown in the figure are the six genes (*exoA*, *exoL*, *exoM*, *exoO*, *exoU*, and *exoW*) that potentially participate in EPS I synthesis in *Sinorhizobium* according to a review (39). Genes involved in the synthesis of other EPSs and other polysaccharides with a putative role in symbiosis (e.g., lipopolysaccharides and capsular polysaccharides) are not included because they are less well characterized (39, 40).
6. Nif genes: The following six genes constitute the minimum gene cluster for nitrogen fixation, namely *nifH*, *nifD*, *nifK*, *nifE*, *nifN* and *nifB* (41, 42). The FeMo nitrogenase consists of two components: a reductase component encoded by *nifH*, and a metallocluster-containing heterotetramer encoded by *nifD* and *nifK*. *nifE*, *nifN*, and *nifB* participate in the synthesis and insertion of the Fe-Mo cofactor into the nitrogenase.
7. Fix genes: *fixLJKT* are involved in regulating the expression of *nif* and many other genes required for growth under microaerobic conditions (43). The *fixABCX* encode products are involved in transferring electrons to nitrogenase (44).
8. Hup genes: The products of *hupL* and *hupS* constitute the central parts of the hydrogenase (45).
9. Denitrification genes: Denitrification from NO_3_^-^ to N_2_ involves multiple steps. *napABC* encode the periplasmic nitrate reductase, which catalyzes the conversion of NO_3_^-^ to NO_2_^-^ (46). Note that *narGHI* encodes a cytoplasm-targeted nitrate reductase, which, however, is not common in rhizobia and thus not shown in the figure (47). *nirK* encodes the nitrite reductase catalyzing the conversion of NO_2_^-^ to NO (48). *norBCD* encodes the nitric oxide reductase converting NO to N_2_O (49). *nosZ* encodes the nitrous oxide reductase converting N_2_O to N_2_, the terminal step in the denitrification pathway (50).
10. PHB (poly-beta-hydroxybutyrate) metabolism: *phaZ*, *phbB* and *phbC* encode the PHB depolymerase (51), the acetoacetyl coenzyme A reductase (52), and the PHB synthase (52), respectively. They are the key genes involved PHB metabolism in the *Rhizobiaceae* (53).
11. Dct: *dctABD* encode the three main components of the Dct transporter, where DctA functions as a dicarboxylate carrier protein and DctBD serve as a two-component kinase regulatory system (54).
12. Phn: Proteins encodes by the operon *phn* are responsible for organophosphonate catabolism. Among all genes located in the operon, *phnGHIJK* encode a complex in the form of PhnG_4_H_2_I_2_J_2_K that is essential for organophosphonate utilization in *E. coli* (55).
13. Ect (ectoine synthesis): *ectABCD* are involved in the biosynthesis of ectoine from the precursor L-aspartate-β-semialdehyde in *E. coli* (56). Only *ectB* and *ectD* were detected as genes significantly correlated with nodule-associated *Rhizobiales*, and were thus shown in the figure as representative genes.
14. Siderophore: *fegA* is reportedly the first gene encoding a putative Fe(III)-siderophore receptor in rhizobia (57).
15. Class Ib RNR (ribonucleotide reductase): *nrdEFHI* encode the four components of the class Ib RNR in bacteria (58).
16. Adhesin: AidA, Hia and TAA are three adhesins identified to be positively correlated with animal associates. They are closely related to T5SS and involved in pathogenesis in bacteria (59).
17. Multidrug resistance: *oprN*, *macA*, *macB* and *sdrM* are identified to be positively correlated with animal associates, and encode components of multidrug efflux pumps (60–62).
18. Nickel transporter: *nikABCDE* encode the nickel-transport system in bacteria (63), which plays a role in its virulence and is involved in the survival in hosts (64, 65).
19. Cobalt transporter: *cbiMNQO* encode the bacterial cobalt-transport system (66), which is likely important to *Brucella* virulence [reviewed in (65)].
20. Amino acid transporters: Shown in the figure are all amino acid transporters that are significantly positively correlated with animal-associated *Rhizobiales*. *cycA*: D-serine/D-alanine/glycine transporter. *tcyP*: L-cystine uptake protein. GAMAperm: GABA permease. gltL-aatP, gltL-aatQ, and gltL-aatM: glutamate/aspartate transport system ATP-binding protein. *hutT*: histidine transporter. *metI* and *metN*: D-methionine ABC transporter.

**Part C. Calibration information**

Calibration I

Node: 1 (Root)

Minimum age: 3,000 Ma

Maximum age: 3,300 Ma

Age justification: We extracted all of the four time estimates from TimeTree (67) for the root, i.e., the divergence time between *Proteobacteria* and cyanobacteria, which ranges from 3100 Mya to 3254 Mya (5, 68–70). Hence, we decided to use 3,300 and 3,000 Ma as the maximum and minimum time constraints of the root, respectively.

Studies using this time constraint: N/A

Calibration II

Node: 2 (crown group of cyanobacteria)

Minimum age: 2,320 Ma

Maximum age: 3,000 Ma

Age justification: The age constraints of the crown group of cyanobacteria were based on the origin of oxygenic photosynthesis. Its lower boundary was set to be 2,320 Ma, which is based on geochemical estimate of the rise of atmospheric oxygen (71). Based on sensitive redox proxies, it is suggested that oxygenic cyanobacteria could have originated from ~3,000 Mya (72), and thus we used it as the upper boundary of this node.

Studies using this time constraint: (73–75)

Calibration III

Node: 3 (crown group of *Nostocales*)

Minimum age: 2,100 Ma

Maximum age: 2,450 Ma

Age justification: The microfossils of Nostocalean akinete (a spore-like structure) likely originated before 2,100 Mya based on fossil records (76, 77). The maximum age for akinete‐containing *Nostocaleans* was set to be 2,450 Ma because massive free oxygen, which favored the origin of akinetes, first appeared during that time (76).

Studies using this time constraint: (75)

Calibration IV

Node: 3 (crown group of *Nostocales*)

Minimum age: 1,600 Ma

Maximum age: 1,900 Ma

Age justification: The use of 2,100 Mya as the minimum age of cyanobacteria has caused disputes because those fossils may represent an early-split cyanobacterial lineage and thus cannot be reliably assigned to *Nostocales* (78, 79). A more conservative minimum age is based on the fossil of akinetes dating back to 1,600 Mya discovered in McArthur Group, northern Australia (80). For the maximum age, according to the studies (81, 82), it was not until ~1,900 Mya that microfossils that have sheaths and increased cell diameters, which are typically characteristic to *Nostocales* and *Pleurocapsales*, started to become popular. Therefore, the maximum age was set to be 1,900 Mya.

Studies using this time constraint: (73, 74)

Calibration V

Node: 4 (crown group of *Pleurocapsales*)

Minimum age: 1,700 Ma

Maximum age: 2,450 Ma

Age justification: That 1,700 Ma was chosen as the minimum age of this group of cyanobacteria was on the basis of the estimated age of the microfossils of *Pleurocapsales* found in Hebei, China, which dates back to ca. 1,700 Mya (83). The ancestor of *Pleurocapsales* is estimated to have large cell diameters. Because some studies suggest that cyanobacteria with large cell diameters likely originated after 2,450 Mya, this time information was used as the maximum age for the *Pleurocapsales* (76, 84).

Studies using this time constraint: (75)

Calibration VI

Node: 4 (crown group of *Pleurocapsales*)

Minimum age: 1,700 Ma

Maximum age: 1,900 Ma

Age justification: The minimum age was set for the same reason as Calibration V. The maximum age was set for the same reason as Calibration IV.

Studies using this time constraint: (73, 74)

Calibration VII

Node: 3 (crown group of *Nostocales*)

Minimum age: -

Maximum age: 2,100 Ma

Age justification: It is proposed that the specialized cells of *Nostocales* (e.g., the heterocyst) evolved in response to atmospheric free oxygen, which, according to a study (76), did not reach the levels allowing for heterocyst differentiation at ca. 2,100 Mya.

Studies using this time constraint: (73)

Calibration VIII

Node: 5 (LCA of Alpha-, Beta-, and Gammaproteobacteria)

Minimum age: 2,360 Ma

Maximum age: 2,620 Ma

Age justification: Due to the lack of fossil records in *Proteobacteria*, we chose the time boundaries of the LCA of Alpha-, Beta, and Gammaproteobacteria based on previous molecular dating studies. There are five estimates deposited in the TimeTree database (67). Four of them consistently inferred the first appearance of this group of bacteria to range from 2,360 to 2,620 Mya (5, 68–70). The remaining study suggests its origin dating back to only 606 Mya (85). This result largely underestimates the origin of the LCA of Alpha-, Beta-, and Gammaproteobacteria because not only it is contradictory to the estimates above, but also the divergence time (29.3 ± 3.0 Mya) of the *Hedysaroid* clade, the current host of a couple of *Rhizobium* strains, was used as the only time constraint in their analysis. By doing so, the authors did not take into consideration the shift of legume hosts for rhizobia, which, in fact, can be very frequent in nature (86–89). If the ancestor of those *Rhizobium* strains nodulated legumes from non-*Hedysaroid* clade and switched to *Hedysaroid* later in evolution, it would plausibly lead to an underestimate of the ages of all analyzed lineages in that study.

Studies using this time constraint: N/A

Calibration IX

Node: 6 (crown group of *Methylobacterium* and *Rhizobiaceae*)

Minimum age: -

Maximum age: 507 Ma

Age justification: We constrained the maxima of the crown groups of *Methylobacterium* and *Rhizobiaceae* to 507 Ma, the estimated maximum origin time of land plants (90). Note that this calibration could lead to circular arguments of the hypotheses we examined, i.e., the co-concurrence of host-associated bacteria and their hosts. However, because of the lack of fossils within the *Rhizobiales* (and even the *Proteobacteria*), this calibration may an alternative way to calibrate the evolutionary timeline.

Studies using this time constraint: -

Calibration X

Node: 6 (crown group of *Azorhizobium*, *Bradyrhizobium*, *Mesorhizobium*, *Rhizobium*, and *Sinorhizobium*)

Minimum age: -

Maximum age: 110 Ma

Age justification: We constrained the maxima of the crown groups of the five nodulating-lineages to 110 Ma, the estimated maximum origin time of legumes (9). Note that this calibration could lead to circular arguments of the hypotheses we examined, i.e., the co-concurrence of host-associated bacteria and their hosts. However, because of the lack of fossils within the *Rhizobiales* (and even the *Proteobacteria*), this calibration may an alternative way to calibrate the evolutionary timeline.

Studies using this time constraint: -

**Supplementary references**

1. Ramanan R, Kim BH, Cho DH, Oh HM, Kim HS. 2016. Algae-bacteria interactions: Evolution, ecology and emerging applications. Biotechnol Adv.

2. Turner SL, Young JPW. 2000. The glutamine synthetases of rhizobia: Phylogenetics and evolutionary implications. Mol Biol Evol 17:309–319.

3. Nascimento FF, dos Reis M, Yang ZH. 2017. A biologist’s guide to Bayesian phylogenetic analysis. Nat Ecol Evol 1:1446–1454.

4. David LA, Alm EJ. 2011. Rapid evolutionary innovation during an Archaean genetic expansion. Nature 469:93–96.

5. Marin J, Battistuzzi FU, Brown AC, Hedges SB. 2017. The Timetree of Prokaryotes: New Insights into Their Evolution and Speciation. Mol Biol Evol 34:437–446.

6. Hedges SB, Kumar S. 2009. The Timetree of Life. OUP Oxford.

7. Lavin M, Herendeen PS, Wojciechowski MF. 2005. Evolutionary rates analysis of leguminosae implicates a rapid diversification of lineages during the tertiary. Syst Biol 54:575–594.

8. Foster CSP, Sauquet H, Van Der Merwe M, McPherson H, Rossetto M, Ho SYW. 2017. Evaluating the impact of genomic data and priors on Bayesian estimates of the angiosperm evolutionary timescale. Syst Biol 66:338–351.

9. Barba-Montoya J, dos Reis M, Schneider H, Donoghue PCJ, Yang Z. 2018. Constraining uncertainty in the timescale of angiosperm evolution and the veracity of a Cretaceous Terrestrial Revolution. New Phytol 218:819–834.

10. Li HT, Yi TS, Gao LM, Ma PF, Zhang T, Yang JB, Gitzendanner MA, Fritsch PW, Cai J, Luo Y, Wang H, van der Bank M, Zhang SD, Wang QF, Wang J, Zhang ZR, Fu CN, Yang J, Hollingsworth PM, Chase MW, Soltis DE, Soltis PS, Li DZ. 2019. Origin of angiosperms and the puzzle of the Jurassic gap. Nat Plants 5:461–470.

11. Magallón S, Gómez-Acevedo S, Sánchez-Reyes LL, Hernández-Hernández T. 2015. A metacalibrated time-tree documents the early rise of flowering plant phylogenetic diversity. New Phytol 207:437–453.

12. Bruneau A, Mercure M, Lewis GP, Herendeen PS. 2008. Phylogenetic patterns and diversification in the caesalpinioid legumes. Botany 86:697–718.

13. dos Reis M, Yang Z. 2019. Bayesian molecular clock dating using genome-scale datasets, p. 309–330. *In* Anisimova, M (ed.), Methods in Molecular Biology. Springer New York, New York, NY.

14. Magallón S, Sanderson MJ. 2001. Absolute diversification rates in angiosperm clades. Evolution (N Y) 55:1762–1780.

15. Herendeen PS, Crane PR. 1992. Early caesalpinioid fruits from the Palaeogene of southern England. Adv Legum Syst Part 4 Foss Rec. Royal Botanic Gardens , Kew .

16. Kjer KM, Ware JL, Rust J, Wappler T, Lanfear R, Jermiin LS, Zhou X, Aspöck H, Aspöck U, Beutel RG, Blanke A, Donath A, Flouri T, Frandsen PB, Kapli P, Kawahara AY, Letsch H, Mayer C, McKenna DD, Meusemann K, Niehuis O, Peters RS, Wiegmann BM, Yeates DK, Von Reumont BM, Stamatakis A, Misof B. 2015. Response to Comment on “phylogenomics resolves the timing and pattern of insect evolution.” Science (80- ) 349:487.

17. Misof B, Liu S, Meusemann K, Peters RS, Donath A, Mayer C, Frandsen PB, Ware J, Flouri T, Beutel RG, Niehuis O, Petersen M, Izquierdo-Carrasco F, Wappler T, Rust J, Aberer AJ, Aspöck U, Aspöck H, Bartel D, Blanke A, Berger S, Böhm A, Buckley TR, Calcott B, Chen J, Friedrich F, Fukui M, Fujita M, Greve C, Grobe P, Gu S, Huang Y, Jermiin LS, Kawahara AY, Krogmann L, Kubiak M, Lanfear R, Letsch H, Li Y, Li Z, Li J, Lu H, Machida R, Mashimo Y, Kapli P, McKenna DD, Meng G, Nakagaki Y, Navarrete-Heredia JL, Ott M, Ou Y, Pass G, Podsiadlowski L, Pohl H, Von Reumont BM, Schütte K, Sekiya K, Shimizu S, Slipinski A, Stamatakis A, Song W, Su X, Szucsich NU, Tan M, Tan X, Tang M, Tang J, Timelthaler G, Tomizuka S, Trautwein M, Tong X, Uchifune T, Walzl MG, Wiegmann BM, Wilbrandt J, Wipfler B, Wong TKF, Wu Q, Wu G, Xie Y, Yang S, Yang Q, Yeates DK, Yoshizawa K, Zhang Q, Zhang R, Zhang W, Zhang Y, Zhao J, Zhou C, Zhou L, Ziesmann T, Zou S, Li Y, Xu X, Zhang Y, Yang H, Wang J, Wang J, Kjer KM, Zhou X. 2014. Phylogenomics resolves the timing and pattern of insect evolution. Science (80- ) 346:763–767.

18. Remigi P, Zhu J, Young JPW, Masson-Boivin C. 2016. Symbiosis within Symbiosis: Evolving Nitrogen-Fixing Legume Symbionts. Trends Microbiol.

19. Wang ET, Tian CF, Chen WF, Young JPW, Chen WX, Wang ET. 2019. Current Systematics of Rhizobia, p. 41–102. *In* Ecology and Evolution of Rhizobia. Springer Singapore, Singapore.

20. Salgado MG, van Velzen R, Nguyen T Van, Battenberg K, Berry AM, Lundin D, Pawlowski K. 2018. Comparative analysis of the nodule transcriptomes of ceanothus thyrsiflorus (rhamnaceae, rosales) and datisca glomerata (Datiscaceae, Cucurbitales). Front Plant Sci 871.

21. Soltis DE, Soltis PS, Morgan DR, Swensen SM, Mullin BC, Dowd JM, Martin PG. 1995. Chloroplast gene sequence data suggest a single origin of the predisposition for symbiotic nitrogen fixation in angiosperms. Proc Natl Acad Sci U S A 92:2647–2651.

22. Griesmann M, Chang Y, Liu X, Song Y, Haberer G, Crook MB, Billault-Penneteau B, Lauressergues D, Keller J, Imanishi L, Roswanjaya YP, Kohlen W, Pujic P, Battenberg K, Alloisio N, Liang Y, Hilhorst H, Salgado MG, Hocher V, Gherbi H, Svistoonoff S, Doyle JJ, He S, Xu Y, Xu S, Qu J, Gao Q, Fang X, Fu Y, Normand P, Berry AM, Wall LG, Ané JM, Pawlowski K, Xu X, Yang H, Spannagl M, Mayer KFX, Wong GKS, Parniske M, Delaux PM, Cheng S. 2018. Phylogenomics reveals multiple losses of nitrogen-fixing root nodule symbiosis. Science (80- ) 361:eaat1743.

23. van Velzen R, Doyle JJ, Geurts R. 2019. A Resurrected Scenario: Single Gain and Massive Loss of Nitrogen-Fixing Nodulation. Trends Plant Sci.

24. van Velzen R, Holmer R, Bu F, Rutten L, van Zeijl A, Liu W, Santuari L, Cao Q, Sharma T, Shen D, Roswanjaya Y, Wardhani TAK, Kalhor MS, Jansen J, Johan van den H, Güngör B, Hartog M, Hontelez J, Verver J, Yang WC, Schijlen E, Repin R, Schilthuizen M, Schranz ME, Heidstra R, Miyata K, Fedorova E, Kohlen W, Bisseling T, Smit S, Geurts R. 2018. Comparative genomics of the nonlegume Parasponia reveals insights into evolution of nitrogen-fixing rhizobium symbioses. Proc Natl Acad Sci U S A 115:E4700–E4709.

25. Aoki S, Ito M, Iwasaki W. 2013. From β- To α-proteobacteria: The origin and evolution of rhizobial nodulation genes nodij. Mol Biol Evol 30:2494–2508.

26. Taulé C, Zabaleta M, Mareque C, Platero R, Sanjurjo L, Sicardi M, Frioni L, Battistoni F, Fabiano E. 2012. New betaproteobacterial Rhizobium strains able to efficiently nodulate Parapiptadenia rigida (Benth.) Brenan. Appl Environ Microbiol 78:1692–1700.

27. Mierzwa B, Wdowiak-Wróbel S, Kalita M, Gnat S, Małek W. 2010. Insight into the evolutionary history of symbiotic genes of Robinia pseudoacacia rhizobia deriving from Poland and Japan. Arch Microbiol 192:341–350.

28. Meade A, Pagel M. 2016. BayesTraits V3 Manual.

29. Roche P, Maillet F, Plazanet C, Debellé F, Ferro M, Truchet G, Promé JC, Dénarié J. 1996. The common nodABC genes of Rhizobium meliloti are host-range determinants. Proc Natl Acad Sci U S A 93:15305–15310.

30. Poole P, Ramachandran V, Terpolilli J. 2018. Rhizobia: From saprophytes to endosymbionts. Nat Rev Microbiol 16:291–303.

31. Wais RJ, Keating DH, Long SR. 2002. Structure-function analysis of nod factor-induced root hair calcium spiking in Rhizobium-legume symbiosis. Plant Physiol 129:211–224.

32. van Rhijn P, Vanderleyden J. 1995. The Rhizobium-plant symbiosis. Microbiol Rev.

33. Eichinger V, Nussbaumer T, Platzer A, Jehl MA, Arnold R, Rattei T. 2016. EffectiveDB - Updates and novel features for a better annotation of bacterial secreted proteins and Type III, IV, VI secretion systems. Nucleic Acids Res 44:D669–D674.

34. Wallden K, Rivera-Calzada A, Waksman G. 2010. Type IV secretion systems: Versatility and diversity in function. Cell Microbiol.

35. Belin BJ, Busset N, Giraud E, Molinaro A, Silipo A, Newman DiK. 2018. Hopanoid lipids: From membranes to plant-bacteria interactions. Nat Rev Microbiol.

36. Nukui N, Minamisawa K, Ayabe SI, Aoki T. 2006. Expression of the 1-aminocyclopropane-1-carboxylic acid deaminase gene requires symbiotic nitrogen-fixing regulator gene nifA2 in Mesorhizobium loti MAFF303099. Appl Environ Microbiol 72:4964–4969.

37. Guinel FC. 2015. Ethylene, a hormone at the center-stage of nodulation. Front Plant Sci.

38. Morrone D, Chambers J, Lowry L, Kim G, Anterola A, Bender K, Peters RJ. 2009. Gibberellin biosynthesis in bacteria: Separate ent-copalyl diphosphate and ent-kaurene synthases in Bradyrhizobium japonicum. FEBS Lett 583:475–480.

39. Skorupska A, Janczarek M, Marczak M, Mazur A, Król J. 2006. Rhizobial exopolysaccharides: Genetic control and symbiotic functions. Microb Cell Fact.

40. Marczak M, Mazur A, Koper P, Żebracki K, Skorupska A. 2017. Synthesis of rhizobial exopolysaccharides and their importance for symbiosis with legume plants. Genes (Basel).

41. Wang L, Zhang L, Liu Z, Zhao D, Liu X, Zhang B, Xie J, Hong Y, Li P, Chen S, Dixon R, Li J. 2013. A Minimal Nitrogen Fixation Gene Cluster from Paenibacillus sp. WLY78 Enables Expression of Active Nitrogenase in Escherichia coli. PLoS Genet 9.

42. Curatti L, Hernandez JA, Igarashi RY, Soboh B, Zhao D, Rubio LM. 2007. In vitro synthesis of the iron-molybdenum cofactor of nitrogenase from iron, sulfur, molybdenum, and homocitrate using purified proteins. Proc Natl Acad Sci U S A 104:17626–17631.

43. Cebolla A, Palomares AJ. 1994. Genetic regulation of nitrogen fixation in Rhizobium meliloti. Microbiologia 10:371–384.

44. Edgren T, Nordlund S. 2004. The fixABCX Genes in Rhodospirillum rubrum Encode a Putative Membrane Complex Participating in Electron Transfer to Nitrogenase. J Bacteriol 186:2052–2060.

45. Casalot L, Rousset M. 2001. Maturation of the [NiFe] hydrogenases. Trends Microbiol.

46. Stewart V, Lu Y, Darwin AJ. 2002. Periplasmic nitrate reductase (NapABC enzyme) supports anaerobic respiration by Escherichia coli K-12. J Bacteriol 184:1314–1323.

47. Torres MJ, Rubia MI, De La Peña TC, Pueyo JJ, Bedmar EJ, Delgado MJ. 2014. Genetic basis for denitrification in Ensifer meliloti. BMC Microbiol 14:142.

48. Braker G, Zhou J, Wu L, Devol AH, Tiedje JM. 2000. Nitrite reductase genes (nirK and nirS) as functional markers to investigate diversity of denitrifying bacteria in pacific northwest marine sediment communities. Appl Environ Microbiol 66:2096–2104.

49. Vaccaro BJ, Thorgersen MP, Lancaster WA, Price MN, Wetmore KM, Poole FL, Deutschbauer A, Arkin AP, Adams MWW. 2016. Determining roles of accessory genes in denitrification by mutant fitness analyses. Appl Environ Microbiol 82:51–61.

50. Wyman M, Hodgson S, Bird C. 2013. Denitrifying alphaproteobacteria from the arabian sea that express nosZ, the gene encoding nitrous oxide reductase, in oxic and suboxic waters. Appl Environ Microbiol 79:2670–2681.

51. Trainer MA, Capstick D, Zachertowska A, Lam KN, Clark SRD, Charles TC. 2010. Identification and characterization of the intracellular poly-3-hydroxybutyrate depolymerase enzyme PhaZ of Sinorhizobium meliloti. BMC Microbiol 10.

52. Kadouri D, Burdman S, Jurkevitch E, Okon Y. 2002. Identification and isolation of genes involved in poly(β-hydroxybutyrate) biosynthesis in Azospirillum brasilense and characterization of a phbC mutant. Appl Environ Microbiol 68:2943–2949.

53. Trainer MA. 2009. Carbon metabolism and desiccation tolerance in the nitrogen-fixing rhizobia *Bradyrhizobium japonicum* and *Sinorhizobium meliloti*. PhD Thesis 1–386.

54. Yurgel SN, Kahn ML. 2004. Dicarboxylate transport by rhizobia. FEMS Microbiol Rev.

55. Jochimsen B, Lolle S, McSorley FR, Nabi M, Stougaard J, Zechel DL, Hove-Jensen B. 2011. Five phosphonate operon gene products as components of a multi-subunit complex of the carbon-phosphorus lyase pathway. Proc Natl Acad Sci U S A 108:11393–11398.

56. Louis P, Galinski EA. 1997. Characterization of genes for the biosynthesis of the compatible solute ectoine from Marinococcus halophilus and osmoregulated expression in Escherichia coli. Microbiology 143:1141–1149.

57. LeVier K, Guerinot M Lou. 1996. The Bradyrhizobium japonicum fegA gene encodes an iron-regulated outer membrane protein with similarity to hydroxamate-type siderophore receptors. J Bacteriol 178:7265–7275.

58. Crona M, Torrents E, Røhr ÅK, Hofer A, Furrer E, Tomter AB, Andersson KK, Sahlin M, Sjöberg BM. 2011. NrdH-redoxin protein mediates high enzyme activity in manganese- reconstituted ribonucleotide reductase from Bacillus anthracis. J Biol Chem 286:33053–33060.

59. Leo JC, Grin I, Linke D. 2012. Type V secretion: Mechanism(S) of autotransport through the bacterial outer membrane. Philos Trans R Soc B Biol Sci.

60. Köhler T, Michéa-Hamzehpour M, Henze U, Gotoh N, Curty LK, Pechère JC. 1997. Characterization of MexE-MexF-OprN, a positively regulated multidrug efflux system of Pseudomonas aeruginosa. Mol Microbiol 23:345–354.

61. Kobayashi N, Nishino K, Yamaguchi A. 2001. Novel macrolide-specific ABC-type efflux transporter in Escherichia coli. J Bacteriol 183:5639–5644.

62. Yamada Y, Hideka KI, Shiota S, Kuroda T, Tsuchiya T. 2006. Gene cloning and characterization of SdrM, a chromosomally-encoded multidrug efflux pump, from Staphylococcus aureus. Biol Pharm Bull 29:554–556.

63. Rowe JL, Starnes GL, Chivers PT. 2005. Complex transcriptional control links NikABCDE-dependent nickel transport with hydrogenase expression in Escherichia coli. J Bacteriol 187:6317–6323.

64. Jubier-Maurin V, Rodrigue A, Ouahrani-Bettache S, Layssac M, Mandrand-Berthelot MA, Köhler S, Liautard JP. 2001. Identification of the nik gene cluster of Brucella suis: Regulation and contribution to urease activity. J Bacteriol 183:426–434.

65. Wattam AR, Foster JT, Mane SP, Beckstrom-Sternberg SM, Beckstrom-Sternberg JM, Dickerman AW, Keim P, Pearson T, Shukla M, Ward D V., Williams KP, Sobral BW, Tsolis RM, Whatmore AM, O’Callaghan D. 2014. Comparative phylogenomics and evolution of the brucellae reveal a path to virulence. J Bacteriol 196:920–930.

66. Bao Z, Qi X, Hong S, Xu K, He F, Zhang M, Chen J, Chao D, Zhao W, Li D, Wang J, Zhang P. 2017. Structure and mechanism of a group-I cobalt energy coupling factor transporter. Cell Res 27:675–687.

67. Kumar S, Stecher G, Suleski M, Hedges SB. 2017. TimeTree: A Resource for Timelines, Timetrees, and Divergence Times. Mol Biol Evol 34:1812–1819.

68. Battistuzzi FU, Feijao A, Hedges SB. 2004. A genomic timescale of prokaryote evolution: Insights into the origin of methanogenesis, phototrophy, and the colonization of land. BMC Evol Biol 4:44.

69. Sheridan PP, Freeman KH, Brenchley JE. 2003. Estimated minimal divergence times of the major bacterial and archaeal phyla. Geomicrobiol J 20:1–14.

70. Battistuzzi FU, Hedges SB. 2009. A major clade of prokaryotes with ancient adaptations to life on land. Mol Biol Evol 26:335–343.

71. Kump LR. 2008. The rise of atmospheric oxygen. Nature 451:277–278.

72. Crowe SA, Døssing LN, Beukes NJ, Bau M, Kruger SJ, Frei R, Canfield DE. 2013. Atmospheric oxygenation three billion years ago. Nature 501:535–538.

73. Sánchez-Baracaldo P. 2015. Origin of marine planktonic cyanobacteria. Sci Rep 5:17418.

74. Sánchez-Baracaldo P, Raven JA, Pisani D, Knoll AH. 2017. Early photosynthetic eukaryotes inhabited low-salinity habitats. Proc Natl Acad Sci U S A 114:E7737–E7745.

75. Sánchez-Baracaldo P, Ridgwell A, Raven JA. 2014. A neoproterozoic transition in the marine nitrogen cycle. Curr Biol 24:652–657.

76. Tomitani A, Knoll AH, Cavanaugh CM, Ohno T. 2006. The evolutionary diversification of cyanobacteria: Molecular-phylogenetic and paleontological perspectives. Proc Natl Acad Sci U S A 103:5442–5447.

77. Knoll AH, Golubic S, Green J, Swett K. 1986. Organically preserved microbial endoliths from the late Proterozoic of East Greenland. Nature 321:856–857.

78. Betts HC, Puttick MN, Clark JW, Williams TA, Donoghue PCJ, Pisani D. 2018. Integrated genomic and fossil evidence illuminates life’s early evolution and eukaryote origin. Nat Ecol Evol 2:1556–1562.

79. Butterfield NJ. 2015. Proterozoic photosynthesis - a critical review, p. 953–972. *In* Palaeontology.

80. GOLUBIC S, SERGEEV VN, KNOLL AH. 1995. Mesoproterozoic Archaeoellipsoidès: akinetes of heterocystous cyanobacteria. Lethaia 28:285–298.

81. Golubic S, Seong-Joo L. 1999. Early cyanobacterial fossil record: Preservation, palaeoenvironments and identification. Eur J Phycol 34:339–348.

82. Sergeev VN, Gerasimenko LM, Zavarzin GA. 2002. The Proterozoic history and present state of cyanobacteria. Microbiology.

83. Zhang Y, Golubic S. 1987. Endolithic microfossils (cyanophyta) from early Proterozoic stromatolites, Hebei, China. Acta Micropalaeontologica Sin 4:1–3.

84. Blank CE, SÁnchez-Baracaldo P. 2010. Timing of morphological and ecological innovations in the cyanobacteria - A key to understanding the rise in atmospheric oxygen. Geobiology 8:1–23.

85. Chriki-Adeeb R, Chriki A. 2016. Estimating divergence times and substitution rates in Rhizobia. Evol Bioinforma 12:87–97.

86. Young JPW, Johnston AWB. 1989. The evolution of specificity in the legume-rhizobium symbiosis. Trends Ecol Evol 4:341–349.

87. Mutch LA, Young JPW. 2004. Diversity and specificity of Rhizobium leguminosarum biovar viciae on wild and cultivated legumes. Mol Ecol 13:2435–2444.

88. Wang D, Yang S, Tang F, Zhu H. 2012. Symbiosis specificity in the legume - rhizobial mutualism. Cell Microbiol.

89. Masson-Boivin C, Giraud E, Perret X, Batut J. 2009. Establishing nitrogen-fixing symbiosis with legumes: how many rhizobium recipes? Trends Microbiol.

90. Morris JL, Puttick MN, Clark JW, Edwards D, Kenrick P, Pressel S, Wellman CH, Yang Z, Schneider H, Donoghue PCJ. 2018. The timescale of early land plant evolution. Proc Natl Acad Sci U S A 115:E2274–E2283.
