# Supplementary figures and images for: Evolutionary Timeline and Genomic Plasticity Underlying the Lifestyle Diversity in Rhizobiales
Source: mSystems. 2020 Jul 14;5(4):e00438-20. doi: 10.1128/mSystems.00438-20 (PMC7363004; doi:10.1128/mSystems.00438-20)

a

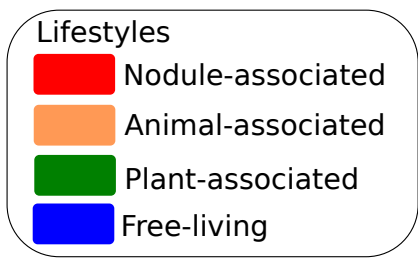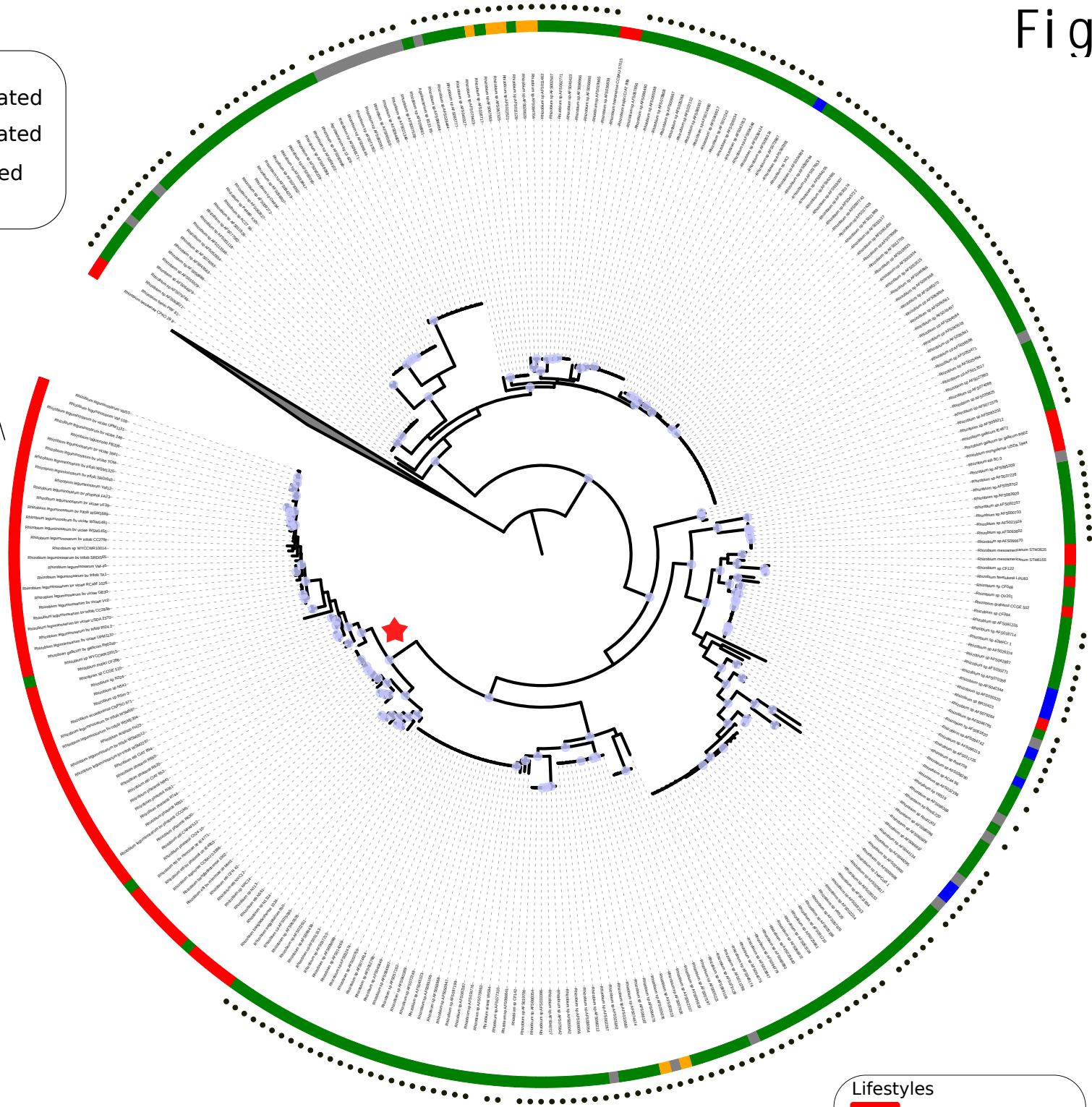

b

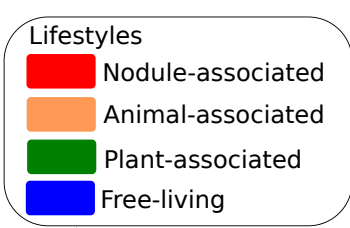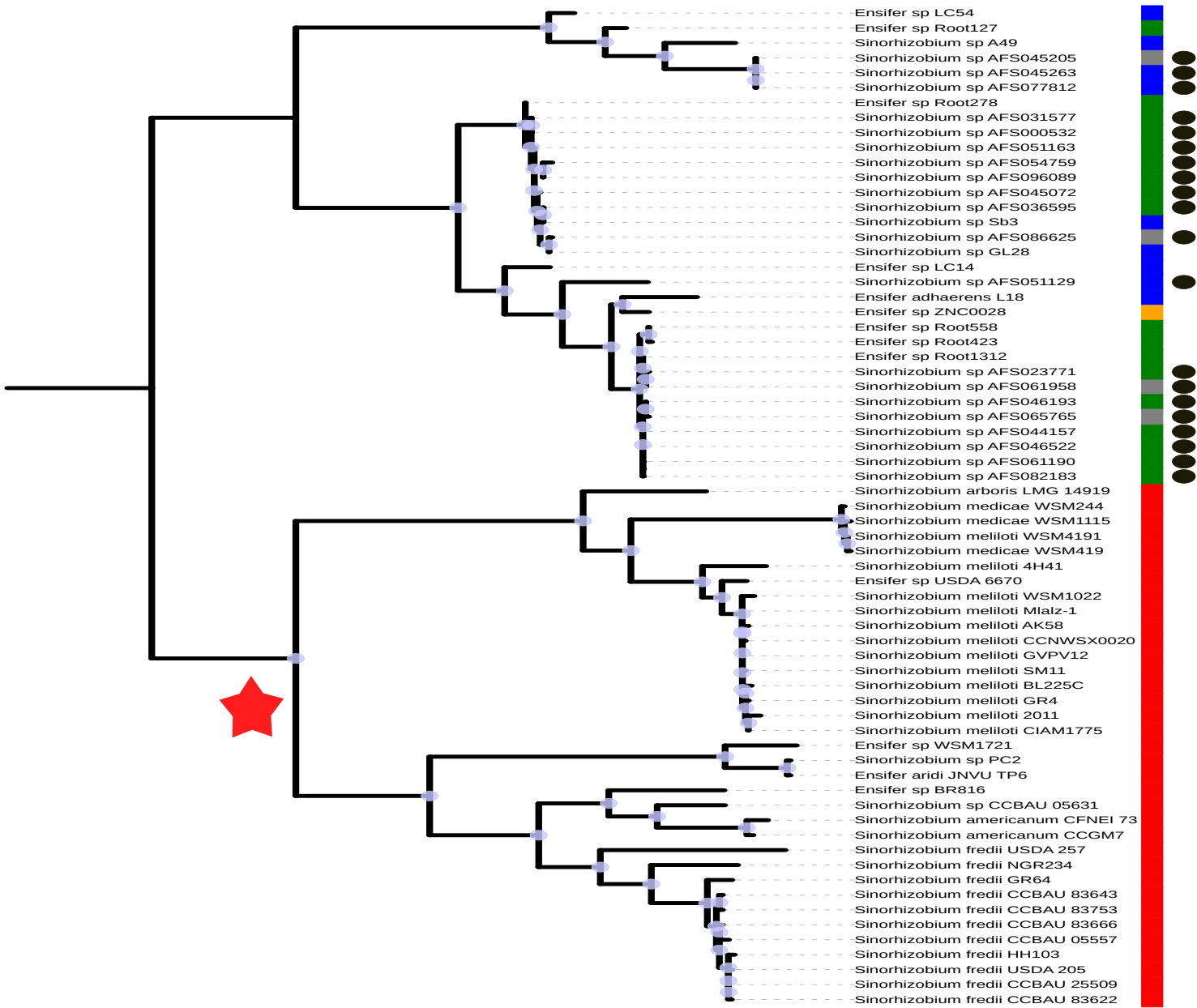

Supplement: FIG S1 [file mSystems.00438-20-sf001.pdf]

## Branch colors

■ Nodule-associated   
 ■ Plant-associated   
 ■ Animal-associated   
 ■ Free-living

**a**

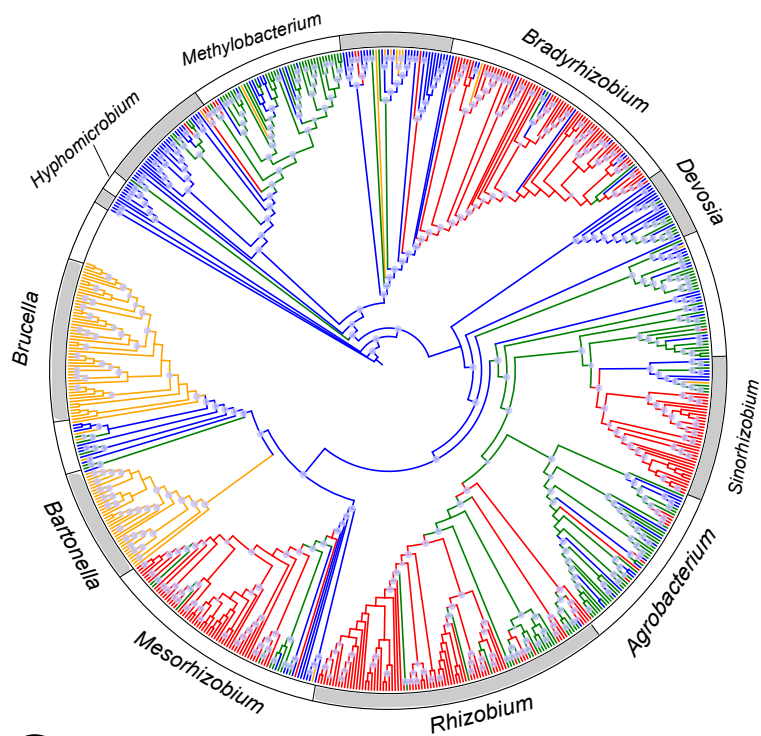

**b**

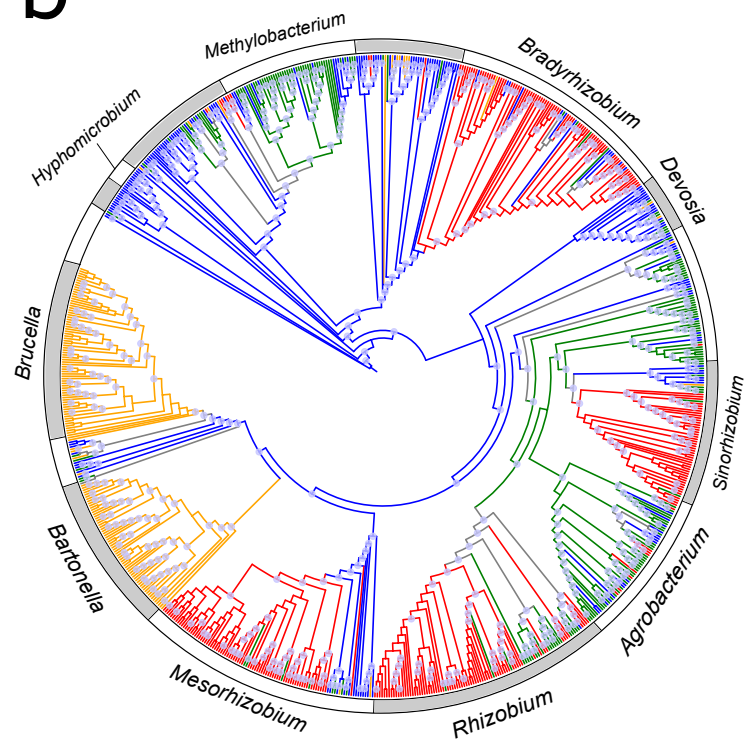

**c**

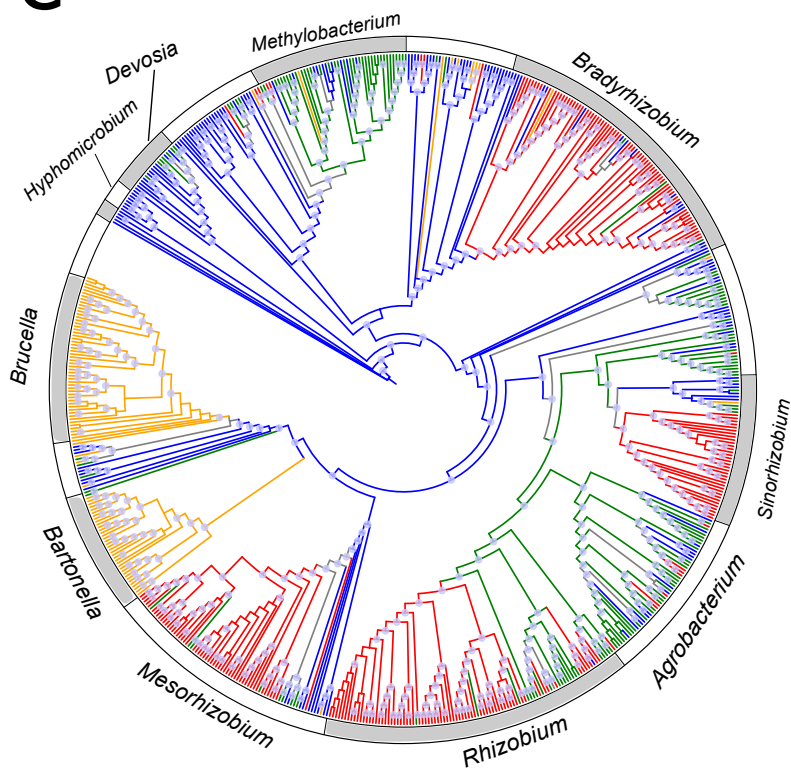

Supplement: FIG S2 [file mSystems.00438-20-sf002.pdf]

Fig. S5

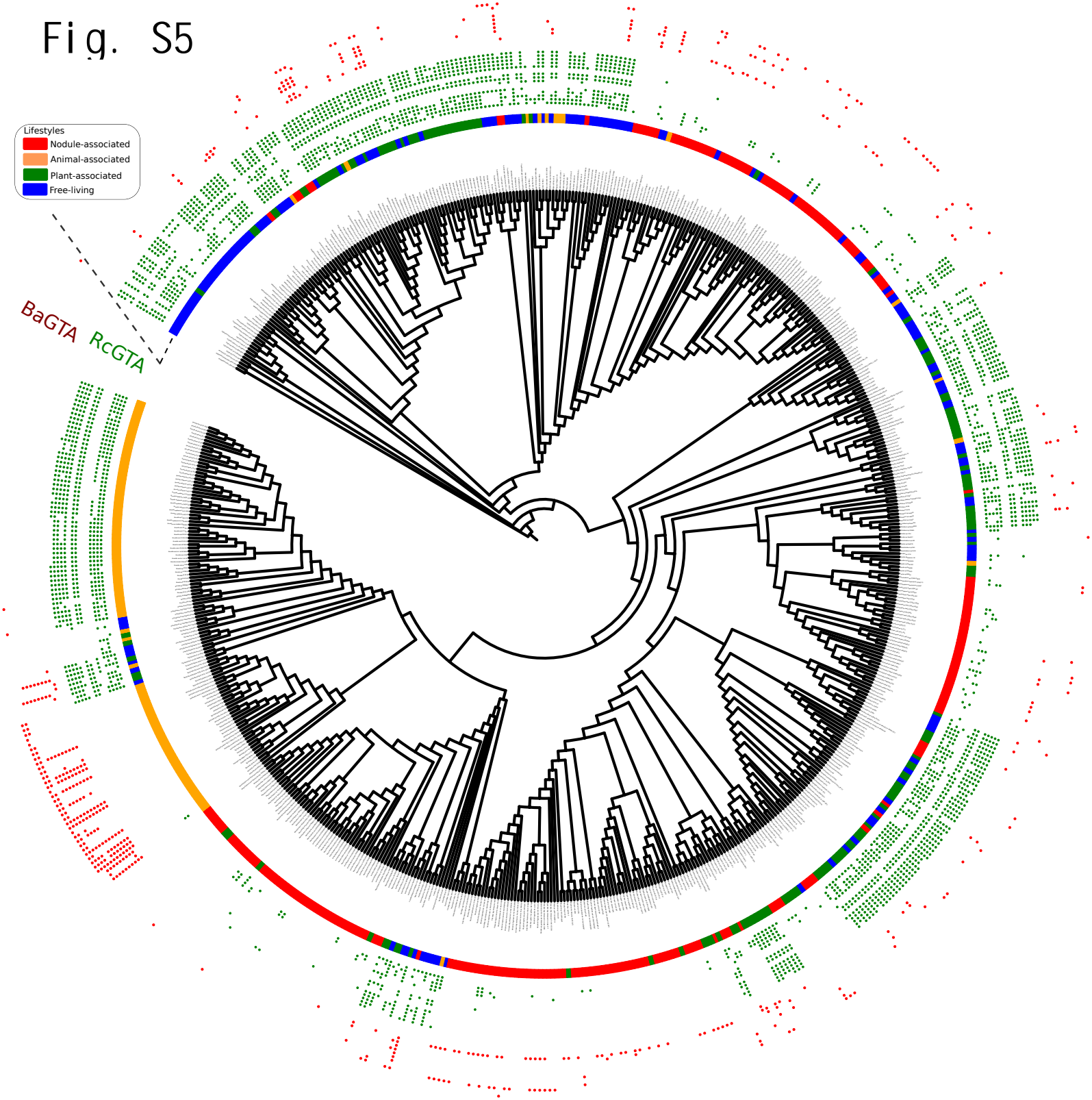

Supplement: FIG S5 [file mSystems.00438-20-sf005.pdf]

Fig. S6

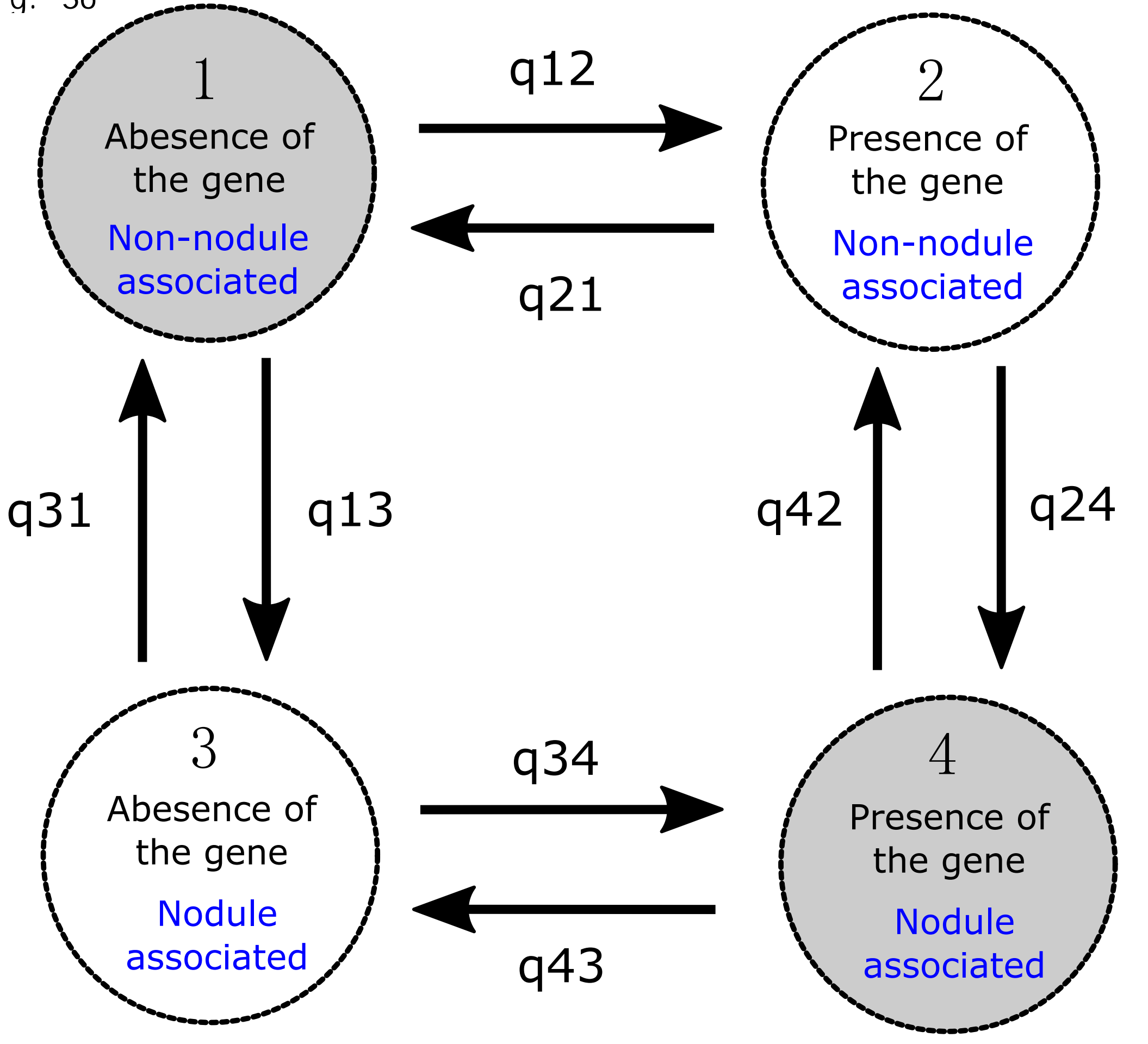

Supplement: FIG S6 [file mSystems.00438-20-sf006.pdf]

Fig. S8

### TIGRFAM

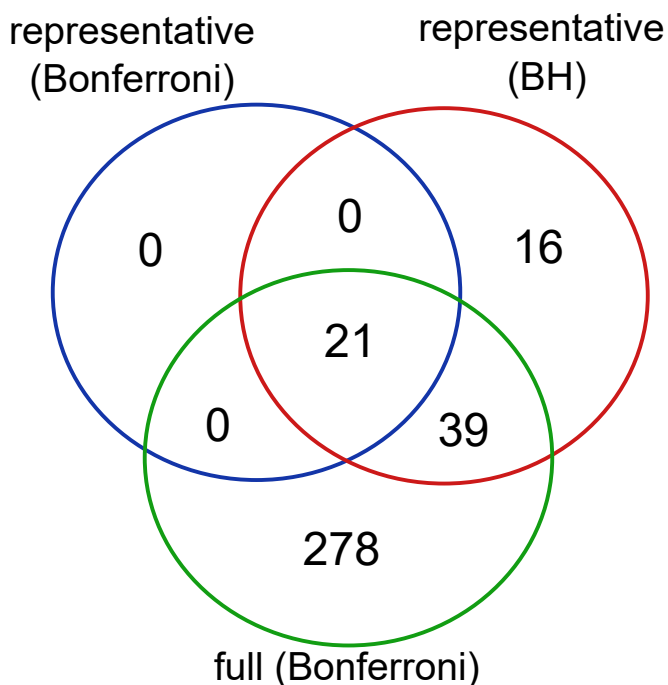

### COG

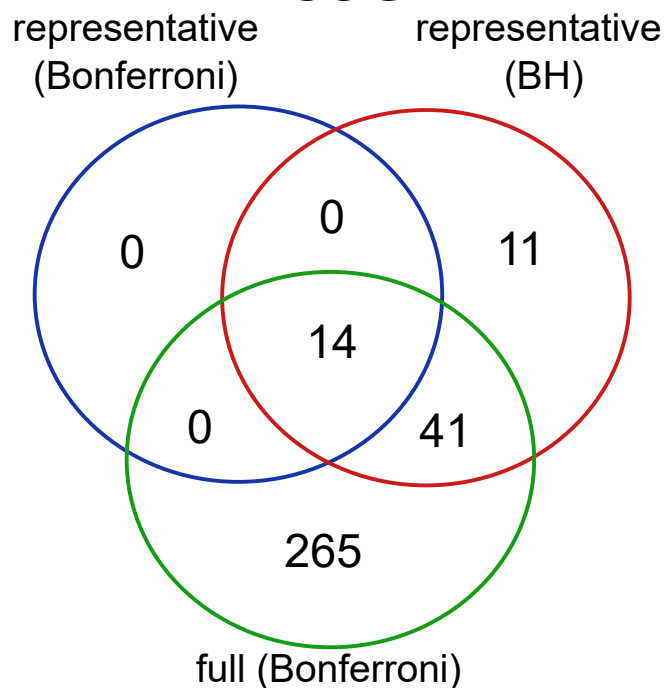

### KEGG

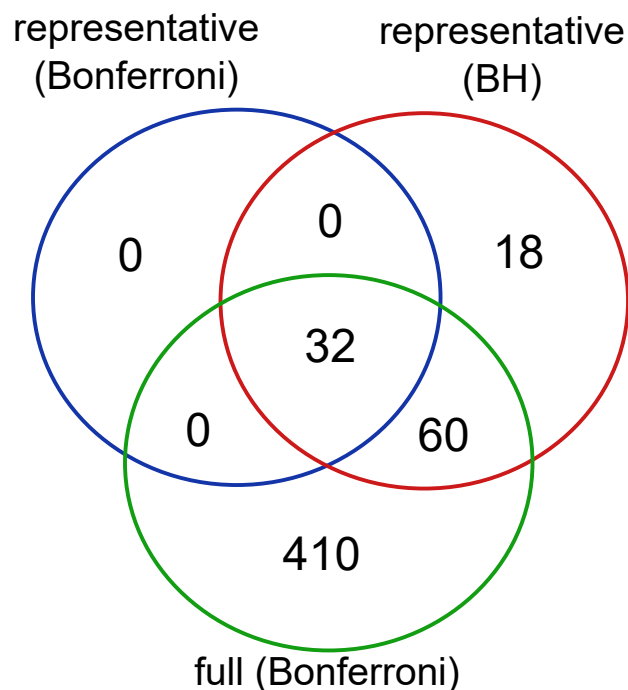

### pfam

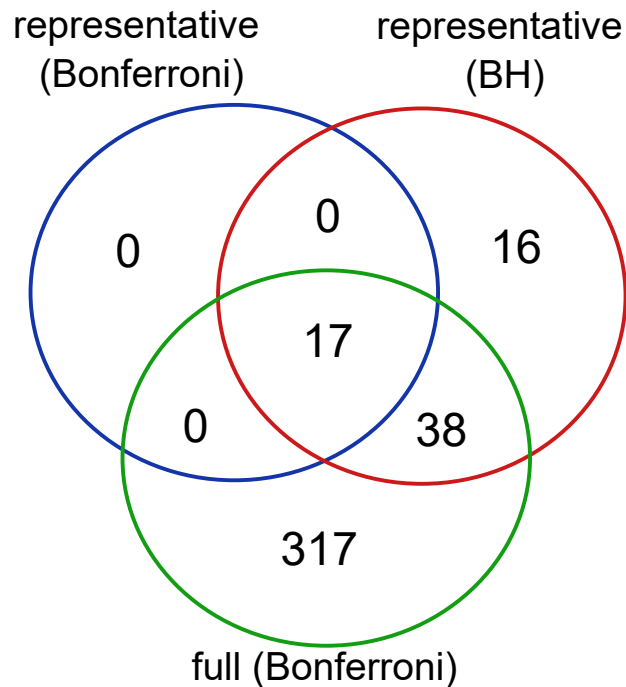

Supplement: FIG S8 [file mSystems.00438-20-sf008.pdf]
