# Supplementary material for: Evolutionary Timeline and Genomic Plasticity Underlying the Lifestyle Diversity in Rhizobiales
Source: mSystems. 2020 Jul 14;5(4):e00438-20. doi: 10.1128/mSystems.00438-20 (PMC7363004; doi:10.1128/mSystems.00438-20)

Fig. S3

a

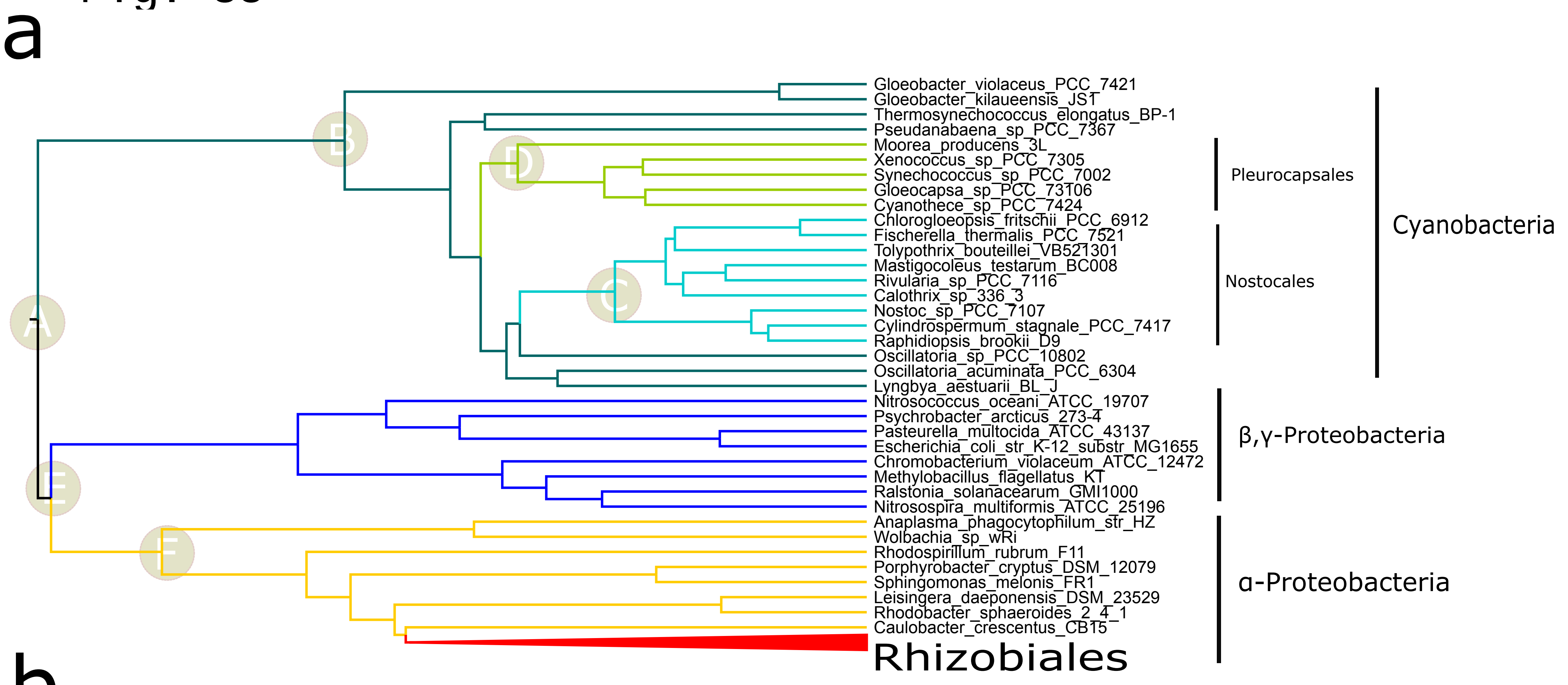

b

	Secondary calibrations				Secondary calibrations		Calibrations based on hosts				
**Set**	**Node 1**	**Node 2**	**Node 3**	**Node 4**	**Node 5**	**Node 6**	**Methylobacterium, Rhizobiaceae**	**Azo-, Brady-, Meso-. Sino-, Rhizobium**	**Time constraints in Note S1**	**Partition scheme**	**Gene sets**
Set 1	3000-3300	2320-3000	1600-1900	1700-1900	2360-2620	1600-2050			I, II, IV, VI, VIII, IX	full	Battistuzzi
Set 2	3000-3300	2320-3000	2100-2450	1700-2450	2360-2620	1600-2050			I, II, III, V, VIII, IX	full	Battistuzzi
Set 3	3000-3300	2320-3000	<2100	1700-1900	2360-2620	1600-2050			I, II, IV, VII, VIII, IX	full	Battistuzzi
Set 4	3000-3300	2320-3000			2360-2620	1600-2050			I, II, VIII, IX	full	Battistuzzi
Set 5	3000-3300	2320-3000	1600-1900	1700-1900					I, II, IV, VI	full	Battistuzzi
Set 6	3000-3300	2320-3000	2100-2450	1700-2450					I, II, III, V	full	Battistuzzi
Set 7	3000-3300	2320-3000	<2100	1700-1900					I, II, IV, VII	full	Battistuzzi
Set 8	3000-3300	2320-3000							I, II	full	Battistuzzi
Set 9	3000-3300	2320-3000	2100-2450	1700-2450			<507		I, II, IV, VI, VIII, IX	full	Battistuzzi
Set 10	3000-3300	2320-3000	2100-2450	1700-2450				<110	I, II, IV, VI, VIII, X	full	Battistuzzi
Set 11	3000-3300	2320-3000	1600-1900	1700-1900	2360-2620	1600-2050			I, II, IV, VI, VIII, IX	ModelFinder	Battistuzzi
Set 12	3000-3300	2320-3000	2100-2450	1700-2450	2360-2620	1600-2050			I, II, IV, VI, VIII, IX	full	OrthoFinder

c

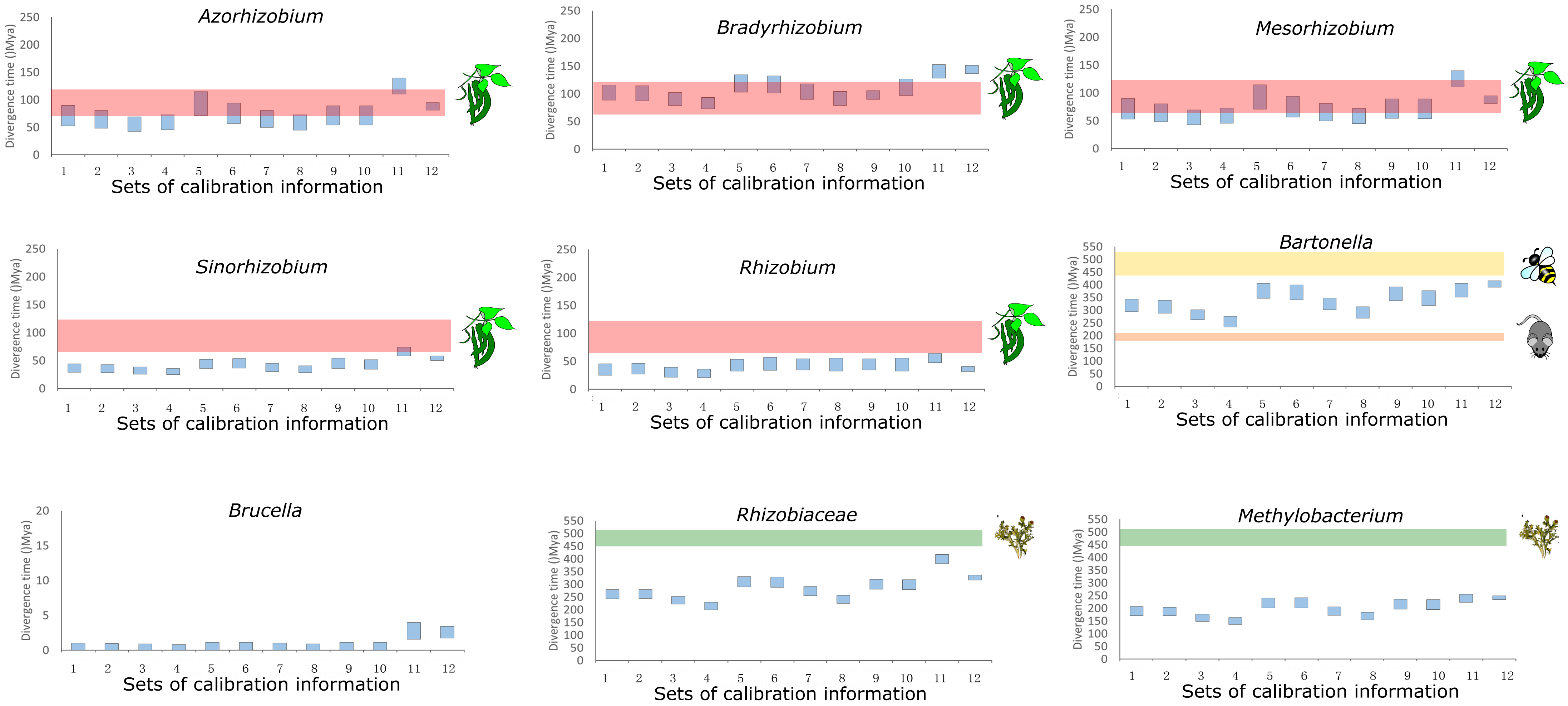

Supplement: FIG S3 [file mSystems.00438-20-sf003.pdf]
