# Supplementary material for: Evolutionary Timeline and Genomic Plasticity Underlying the Lifestyle Diversity in Rhizobiales
Source: mSystems. 2020 Jul 14;5(4):e00438-20. doi: 10.1128/mSystems.00438-20 (PMC7363004; doi:10.1128/mSystems.00438-20)
Supplement: FIG S4 [file mSystems.00438-20-sf004.pdf]

Fig. S4

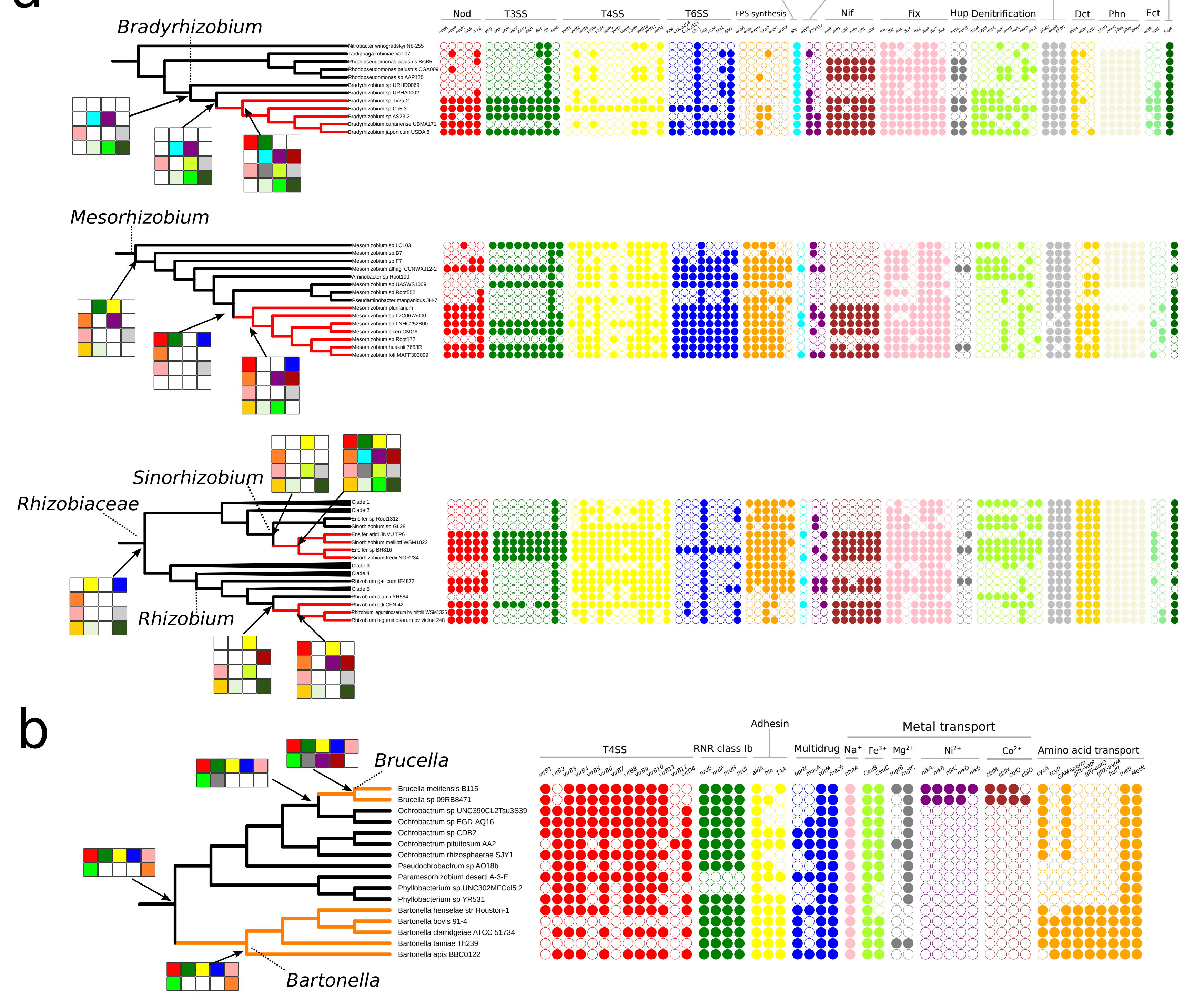

a
Haponoid synthesis
Plant horhomone synthesis
PHB metabolism
Siderophore
Nod
T3SS
T4SS
T6SS
EPS synthesis
Nif
Fix
Hup
Denitrification
Dct
Phn
Ect
Bradyrhizobium
Nitrobacter winogradskyi Nb-255
Tardiphaga robiniae Vaf-07
Rhodopseudomonas palustris BisB5
Rhodopseudomonas palustris CGA009
Rhodopseudomonas sp AAP120
Bradyrhizobium sp URHD0069
Bradyrhizobium sp URHA0002
Bradyrhizobium sp Tv2a-2
Bradyrhizobium sp Cp5 3
Bradyrhizobium sp AS23 2
Bradyrhizobium canariense UBMA171
Bradyrhizobium japonicum USDA 6
Mesorhizobium
Mesorhizobium sp LC103
Mesorhizobium sp B7
Mesorhizobium sp F7
Mesorhizobium alhagi CCNWXJ12-2
Aminobacter sp Root100
Mesorhizobium sp UASWS1009
Mesorhizobium sp Root552
Pseudaminobacter manganicus JH-7
Mesorhizobium plurifarium
Mesorhizobium sp L2C067A000
Mesorhizobium sp LNHC252B00
Mesorhizobium ciceri CMG6
Mesorhizobium sp Root172
Mesorhizobium huakuii 7653R
Mesorhizobium loti MAFF303099
Sinorhizobium
Rhizobiaceae
Rhizobium
Clade 1
Clade 2
Ensifer sp Root1312
Sinorhizobium sp GL28
Ensifer aridi JNVU TP6
Sinorhizobium meliloti WSM1022
Ensifer sp BR816
Sinorhizobium fredii NGR234
Clade 3
Clade 4
Rhizobium gallicum IE4872
Clade 5
Rhizobium alamii YR584
Rhizobium etli CFN 42
Rhizobium leguminosarum bv trifolii WSM1325
Rhizobium leguminosarum bv viciae 248
b
Adhesin
Metal transport
T4SS
RNR class Ib
Multidrug
Na+
Fe3+
Mg2+
Ni2+
Co2+
Amino acid transport
virB1
virB2
virB3
virB4
virB5
virB6
virB7
virB8
virB9
virB10
virB11
virB12
virD4
nrdE
nrdF
nrdH
nrdI
aidA
hia
TAA
oprN
macA
sdrM
macB
nhaA
CeuB
CeuC
mgtB
mgtC
nikA
nikB
nikC
nikD
nikE
cbiM
cbiN
cbiQ
cbiO
cycA
tcyP
GAMAperm
gltL-aatP
glt-aatQ
gltK-aatM
hutT
metI
MetN
Brucella
Bartonella
Brucella melitensis B115
Brucella sp 09RB8471
Ochrobactrum sp UNC390CL2Tsu3S39
Ochrobactrum sp EGD-AQ16
Ochrobactrum sp CDB2
Ochrobactrum pituitosum AA2
Ochrobactrum rhizosphaerae SJY1
Pseudochrobactrum sp AO18b
Paramesorhizobium deserti A-3-E
Phyllobacterium sp UNC302MFCol5 2
Phyllobacterium sp YR531
Bartonella henselae str Houston-1
Bartonella bovis 91-4
Bartonella clarridgeiae ATCC 51734
Bartonella tamiae Th239
Bartonella apis BBC0122
