# Supplementary material for: Evolutionary Timeline and Genomic Plasticity Underlying the Lifestyle Diversity in Rhizobiales
Source: mSystems. 2020 Jul 14;5(4):e00438-20. doi: 10.1128/mSystems.00438-20 (PMC7363004; doi:10.1128/mSystems.00438-20)
Supplement: FIG S7 [file mSystems.00438-20-sf007.pdf]

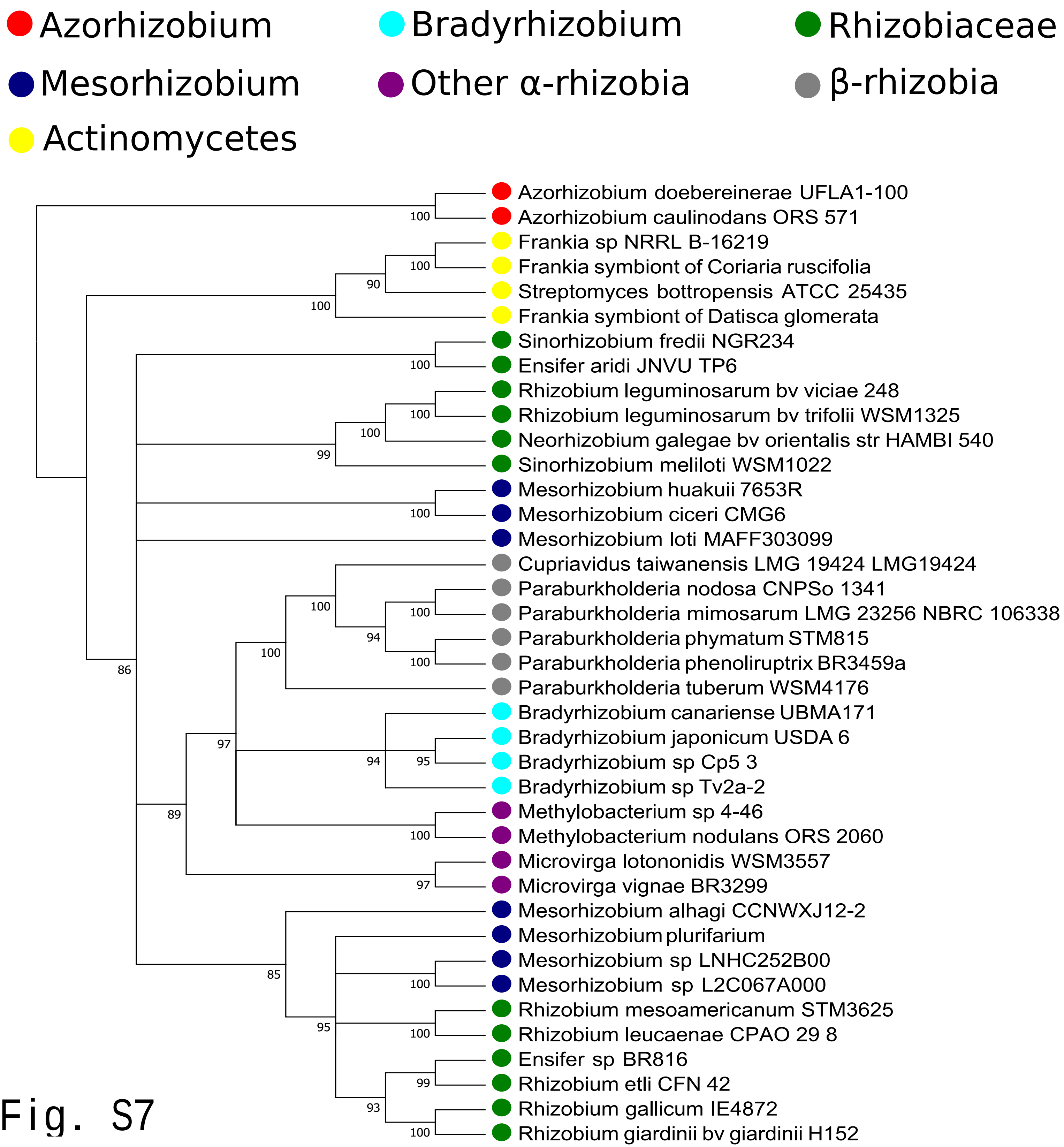
Azorhizobium
Bradyrhizobium
Rhizobiaceae
Mesorhizobium
Other α-rhizobia
β-rhizobia
Actinomycetes
Azorhizobium doebereinerae UFLA1-100
Azorhizobium caulinodans ORS 571
Frankia sp NRRL B-16219
Frankia symbiont of Coriaria ruscifolia
Streptomyces bottropensis ATCC 25435
Frankia symbiont of Datisca glomerata
Sinorhizobium fredii NGR234
Ensifer aridi JNVU TP6
Rhizobium leguminosarum bv viciae 248
Rhizobium leguminosarum bv trifolii WSM1325
Neorhizobium galegae bv orientalis str HAMBI 540
Sinorhizobium meliloti WSM1022
Mesorhizobium huakuii 7653R
Mesorhizobium ciceri CMG6
Mesorhizobium loti MAFF303099
Cupriavidus taiwanensis LMG 19424 LMG19424
Paraburkholderia nodosa CNPSo 1341
Paraburkholderia mimosarum LMG 23256 NBRC 106338
Paraburkholderia phymatum STM815
Paraburkholderia phenoliruptrix BR3459a
Paraburkholderia tuberum WSM4176
Bradyrhizobium canariense UBMA171
Bradyrhizobium japonicum USDA 6
Bradyrhizobium sp Cp5 3
Bradyrhizobium sp Tv2a-2
Methylobacterium sp 4-46
Methylobacterium nodulans ORS 2060
Microvirga lotononidis WSM3557
Microvirga vignae BR3299
Mesorhizobium alhagi CCNWXJ12-2
Mesorhizobium plurifarium
Mesorhizobium sp LNHC252B00
Mesorhizobium sp L2C067A000
Rhizobium mesoamericanum STM3625
Rhizobium leucaenae CPAO 29 8
Ensifer sp BR816
Rhizobium etli CFN 42
Rhizobium gallicum IE4872
Rhizobium giardinii bv giardinii H152
100
100
90
100
100
100
100
99
100
86
100
100
94
100
100
97
94
95
89
100
97
85
100
95
100
99
93
100

Fig. S7
